# Global patterns of genomic and phenotypic variation in the invasive harlequin ladybird

**DOI:** 10.1186/s12915-023-01638-7

**Published:** 2023-06-19

**Authors:** Hongran Li, Yan Peng, Yansong Wang, Bryce Summerhays, Xiaohan Shu, Yumary Vasquez, Hannah Vansant, Christy Grenier, Nicolette Gonzalez, Khyati Kansagra, Ryan Cartmill, Edison Ryoiti Sujii, Ling Meng, Xuguo Zhou, Gábor L. Lövei, John J. Obrycki, Arun Sethuraman, Baoping Li

**Affiliations:** 1 Department of Entomology, College of Plant Protection, Nanjing Agricultural University, Nanjing, People’s Republic of China; 2 Shenzhen Branch, Guangdong Laboratory of Lingnan Modern Agriculture, Genome Analysis Laboratory of the Ministry of Agriculture and Rural Affairs, Agricultural Genomics Institute at Shenzhen, Chinese Academy of Agricultural Sciences, Shenzhen, People’s Republic of China; 3 Department of Biological Sciences, California State University, San Marcos, CA USA; 4 Department of Life and Environmental Sciences, University of California, Merced, CA USA; 5 Empresa Brasileira de Pesquisa Agropecuária (Embrapa), Brasilia, DF Brazil; 6 Department of Entomology, University of Kentucky, Lexington, KY USA; 7 Department of Agroecology, Flakkebjerg Research Centre, Aarhus University, Aarhus, Denmark; 8 ELKH-DE Anthropocene Ecology Research Group, University of Debrecen, Debrecen, Hungary; 9 Department of Zoology & Ecology, Hungarian University of Agriculture & Life Sciences, Godollo, Hungary; 10 Department of Biology, San Diego State University, San Diego, CA USA

**Keywords:** Invasion biology, *mtCOI*, Evolutionary history, Adaptation, Life history, Population genomics

## Abstract

**Background:**

The harlequin ladybird *Harmonia axyridis* (Coleoptera: Coccinellidae), native to Asia, has been introduced to other major continents where it has caused serious negative impacts on local biodiversity. Though notable advances to understand its invasion success have been made during the past decade, especially with then newer molecular tools, the conclusions reached remain to be confirmed with more advanced genomic analyses and especially using more samples from larger geographical regions across the native range. Furthermore, although *H. axyridis* is one of the best studied invasive insect species with respect to life history traits (often comparing invasive and native populations), the traits responsible for its colonization success in non-native areas warrant more research.

**Results:**

Our analyses of genome-wide nuclear population structure indicated that an eastern Chinese population could be the source of all non-native populations and revealed several putatively adaptive candidate genomic loci involved in body color variation, visual perception, and hemolymph synthesis. Our estimates of evolutionary history indicate (1) asymmetric migration with varying population sizes across its native and non-native range, (2) a recent admixture between eastern Chinese and American populations in Europe, (3) signatures of a large progressive, historical bottleneck in the common ancestors of both populations and smaller effective sizes of the non-native population, and (4) the southwest origin and subsequent dispersal routes within its native range in China. In addition, we found that while two mitochondrial haplotypes-Hap1 and Hap2 were dominant in the native range, Hap1 was the only dominant haplotype in the non-native range. Our laboratory observations in both China and USA found statistical yet slight differences between Hap1 and Hap2 in some of life history traits.

**Conclusions:**

Our study on *H*. *axyridis* provides new insights into its invasion processes into other major continents from its native Asian range, reconstructs a geographic range evolution across its native region China, and tentatively suggests that its invasiveness may differ between mitochondrial haplotypes.

**Supplementary Information:**

The online version contains supplementary material available at 10.1186/s12915-023-01638-7.

## Background

Invasive species have caused severe, mostly negative impacts on biodiversity and ecosystems globally and led to immeasurable losses to economies and ecosystems [1, 2]. With climate change and globalization, biological invasions are continuing apace [3] and have become an important element of global change [4]. Thus, management and prevention of invasive species have become major long-term challenges [5–7].

The field of invasion biology has long been seeking an understanding of traits that make a species invasive and thus predicable. These traits encompass a suite of interacting phenotypes, such as growth, reproduction, dispersal, and defense against predators, collectively termed the “invasion syndrome” [8–10]. An understanding of evolutionary processes that promote invasion success is essential to illustrate the concept of an invasion syndrome, and thus to develop sound, long-term approaches to preventing future invasions, and to manage extant ones [11, 12]. High-throughput sequencing is now widely appreciated as an important tool for monitoring, managing, and mitigating the impact of invasive species [13–15]. Population genomics can be used to obtain a detailed knowledge of the invasion history, including assessing source populations, routes of spread, number of independent introductions, the effects of genetic bottlenecks and admixture on the establishment success, adaptive potential, and further spread [16–18]. Genome-wide rapid rates of gene loss and gain via duplications in detoxification, gustatory, and chemosensory receptor gene families are also common in some invasive insects [19–22]. Novel allelic combinations due to extensive hybridization have led to increased hybrid fitness and enhanced environmental tolerance in invasive species [23–25]. Similarly, novel mutations, selective sweeps, and rapid fixation in new environments resulting in insecticide and parasite resistance have also been reported [26, 27]. In addition, serial bottleneck effects and reduced genomic diversity, by accelerating the pace of genetic drift and local adaptations to novel environments, may lead to successful invasion [12, 28, 29].

A powerful biological model for studying rapid evolution of phenotypic traits associated with invasions is provided by the invasive harlequin ladybird *Harmonia axyridis* (Coleoptera: Coccinellidae). It is widely distributed in its native eastern Asian range and generally appreciated as an effective natural enemy suppressing populations of aphids and other agricultural pests [30]. Therefore, it was intentionally introduced as a biological control agent to North America and therefrom to Europe and other continents [9, 31, 32]. Its rapid establishment and wide geographical dispersal cause serious concerns for the threat it poses to biodiversity as a generalist predator [33, 34]. The past two decades have seen considerable research effort invested to understand its global invasion, with a special focus on the traits that promote its invasion success [35, 36]. Numerous phenotypic traits, especially life history traits, have been suggested as causal factors, including larger body size, increased physiological defenses against pathogens and predators, opportunistic predation, shorter development time and pre-oviposition period, higher reproductive potential, and higher temperature tolerance [10, 37, 38]. Analyses of mitochondrial [39] and 18 microsatellite nuclear loci [40–42] have suggested (1) complex population structure and heterogeneity between western and eastern North American populations, (2) multiple introductions and bottlenecks prior to establishment in the western and eastern USA, (3) subsequent colonization and establishment of South American and European populations from an eastern north American source, and (4) genetically admixed populations within Europe composed of genes originating from eastern North America, a European biocontrol population (which itself was founded from individuals from China), and western North America. These conclusions remain to be confirmed using genome-scale data which may provide new insights into the invasion success of *H. axyridis*. Furthermore, though constant efforts have been put into understanding *H. axyridis* life history traits that contribute to its invasion success, few studies have directly compared life history traits of genotypes within and between their native and non-native ranges [30].

Here, we present the most comprehensive global range-wide analysis of the nuclear and mitochondrial population genomics of *H. axyridis*. Specifically, we describe (1) global movement patterns using mitochondrial and genome-wide nuclear loci, (2) genome-wide patterns of selection signals to novel non-native environments, (3) genomic diversity, differentiation, and evolutionary demographic history, and (4) fitness differentials between native (eastern and western China) versus non-native (America) mitochondrial haplotypes. Our insights from this combination of genomic and ecological investigations improve our understanding of the invasion success of *H. axyridis* worldwide.

## Results

### Haplotype identification by mtCOI sequencing

We amplified *mtCOI* gene fragments from 1025 *H. axyridis* individuals sampled from native (China) and non-native (North and South America, Europe) regions, obtaining a 620-bp fragment multiple sequence alignment without insertions, deletions, or stop codons. The sequencing yielded 22 haplotypes with at least two identical sequences (Additional file 1: Table S1).

**Fig. 1:**
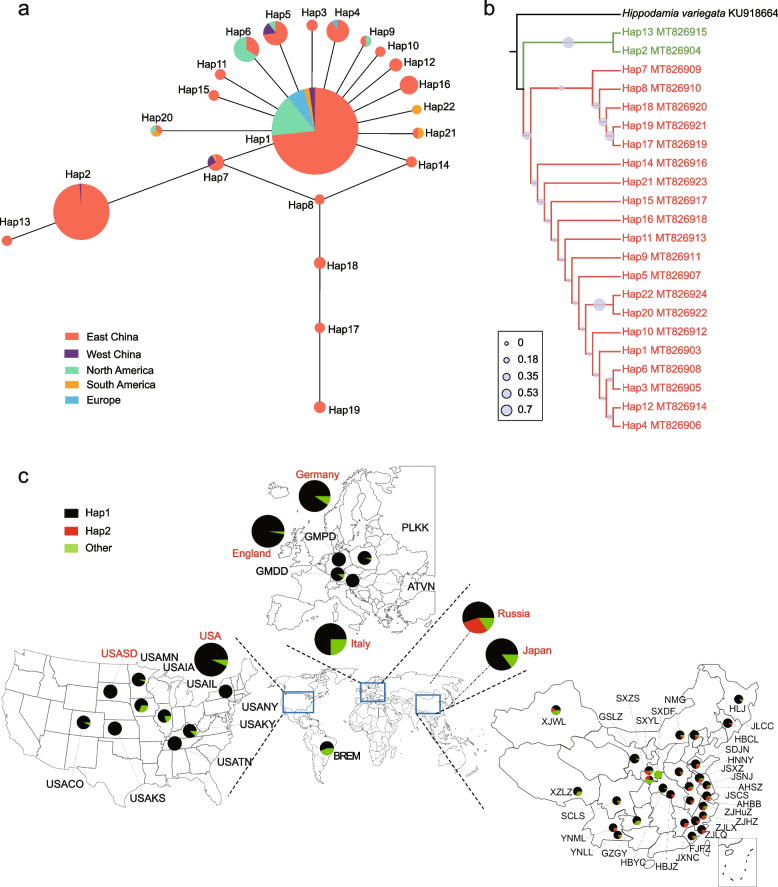
*Harmonia axyridis mtCOI* haplotype phylogeny and distribution across its native and non-native regions. **a** Haplotype network of *H. axyridis* populations across the world, inferred using median-joining algorithm and colored by geography. **b** Phylogenetic tree of *H. axyridis mtCOI* haplotypes using neighbor-joining method, with the grey circles representing the bootstrap value, the polymorphic nucleotide positions of 22 haplotypes were shown in Table S1. **c** Global distribution of *H. axyridis mtCOI* haplotypes, with the country names in red representing a total of 566 *mtCOI* gene sequences downloaded from NCBI database including non-native regions of Germany (42 sequences, 658 bp), Canada (331 sequences, 563 bp), USA (61 sequences, 566 bp), Italy (20 sequences, 566 bp), and the UK (34 sequences, 566 bp), as well as native regions of Russia (58 sequences, 566 bp) and Japan (20 sequences, 566 bp)

Higher mitochondrial haplotypic diversity was shown in *H. axyridis* populations in the native range in China (*H*_d_ = 0.458, calculated after combining data of all the samples; *π* = 0.00204) and the non-native range in South America (*H*_d_ = 0.689; *π* = 0.00235), while lower diversity was found in North American (*H*_d_ = 0.370; *π* = 0.00183) and European populations (*H*_d_ = 0.147; *π* = 0.00048) (Additional file 1: Table S2). Moderate genetic differentiation was detected between native and non-native regions, based on among-site* F*_ST_ values (Additional file 1: Table S3). Haplotype network analysis indicated that mitochondrial haplotypes had a starlike pattern with Hap1 being ancestral and dominant and Hap2 subordinate (Fig. 1a). This pattern was congruent with the phylogenetic tree, in which all 22 haplotypes were clustered into two independent clades (Fig. 1b). The analysis, in combination with all the *mtCOI* sequences that have been deposited in the NCBI database, indicates that Hap1 is dominant, and Hap2 absent in the invaded regions of *H. axyridis* (Fig. 1c).

### Genomic diversity and differentiation

**Fig. 2:**
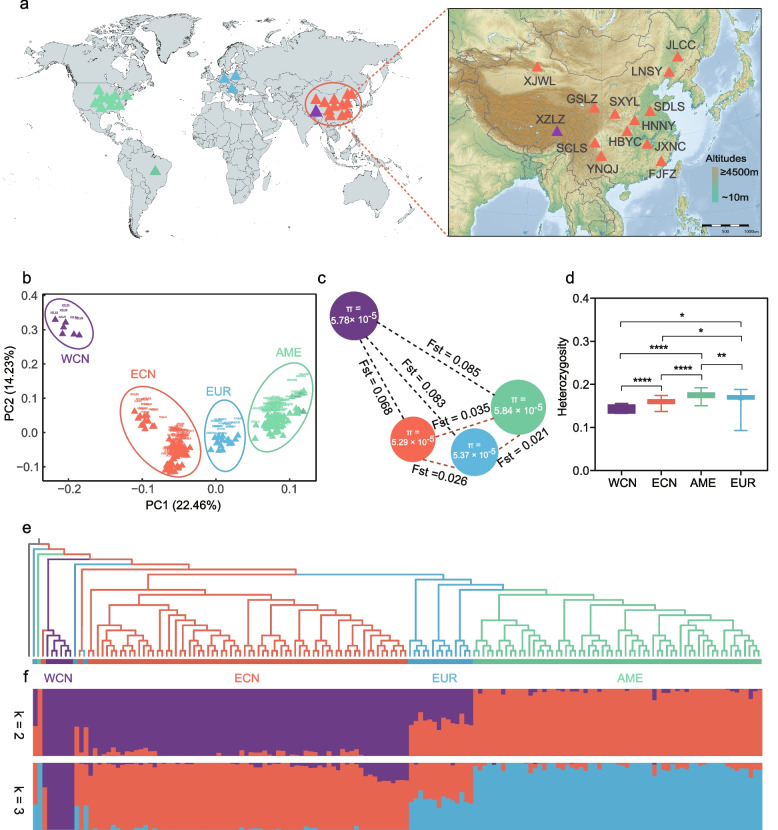
Global geographic location and genetic population structure of *Harmonia axyridis* sample populations divided into four major groups (super-populations) represented by colors of purple for the west China (WCN), red for the east China (ECN), blue for the Europe (EUR), and green for the America (AME). **a** Global distribution of all sampled populations, see Additional file 1: Table S10 for detailed information about ladybird samples collected across the world. **b** A biplot of the first two principle components obtained from the principal component analysis of all sampled populations clustered into four major groups. **c** Genetic diversity (*π*) and differentiation (*F*_ST_) across major groups, with the radius of a circle proportional to the genetic diversity and the length of connecting dashed line to the *F*_ST_ value between two groups. **d** Comparison of individual heterozygosity rates between the major groups, see additional file 1: Table S4 for detailed information about the heterozygosity rate. **e** A phylogenetic tree of all sampled populations, with *Agrilus planipennis* as the outgroup and WCN group as the closest to the outgroup. **f** Map of the structure (*K* = 2 and 3) matching the phylogenetic tree, with different colors representing the components from the respective major groups in each sample population

Sequencing 2b-RAD libraries produced 1230.71 million raw reads from 159 individuals with an average of 7.74 million reads with restriction sites per individual (Fig. 2a). After quality filtering and variant calling using the *H. axyridis* reference genome, we identified 7824 polymorphic SNPs for the further analysis. A principal component analysis (PCA) revealed four putative super-populations corresponding to sample populations from west China (WCN), east China (ECN), Americas (AME), and Europe (EUR). The two principal components accounted for 36.69% of the total variance with the first (PC1) differentiating ECN, AME, and EUR and the second (PC2) distinguishing WCN from the others (Fig. 2b).

To assess the genetic differentiation among the putative super-populations, we measured genetic distances with Weir and Cockerham’s *F*_ST_ between the native (WCN and ECN) and non-native super-populations (AME and EUR) (Fig. 2c). The* F*_ST_ between WCN and AME was 0.085, and between WCN and EUR 0.083, both being higher than *F*_ST_ between ECN and AME (0.035) or between ECN and EUR (0.026); the *F*_ST_ between the WCN and ECN was 0.068. The genome-wide nucleotide diversity (*π*) in both native and non-native ranges indicated little genomic diversity within WCN (*π* = 5.78 × 10^−5^), ECN (*π* = 5.29 × 10^−5^), AME (*π* = 5.84 × 10^−5^), and EUR (*π* = 5.37 × 10^−5^) (Fig. 2c). The heterozygosity estimates within non-native ranges (AME: *Ho* = 0.175; EUR: *Ho* = 0.165) were higher than that within the native ranges (WCN: *Ho* = 0.143; ECN: *Ho* = 0.159) (Fig. 2d; Additional files 1: Table S4).

To further establish the phylogenetic relationship among all 27 geographic populations sampled across the world, we constructed a neighbor-joining phylogenetic tree, using *Agrilus planipennis* (Coleoptera: Buprestidae) as an outgroup. All sample populations clustered into four independent clades corresponding to WCN, ECN, AME and EUR super-populations as deduced by the PCA analysis (Fig. 2e). These super-populations were also supported by population structure analysis using ADMIXTURE (Fig. 2f). When using the minimum cross validation (CV) error value (*K* = 2), we found that the *H. axyridis* populations from China and America exhibited significant population structure, while those from Europe had shared ancestry with the Chinese and American populations. When using K of 3, WCN, ECN, AME, and EUR super-populations could be readily distinguished (Fig. 2f; Additional file 2: Fig. S1a).

### Population origin and expansion within China

**Fig. 3:**
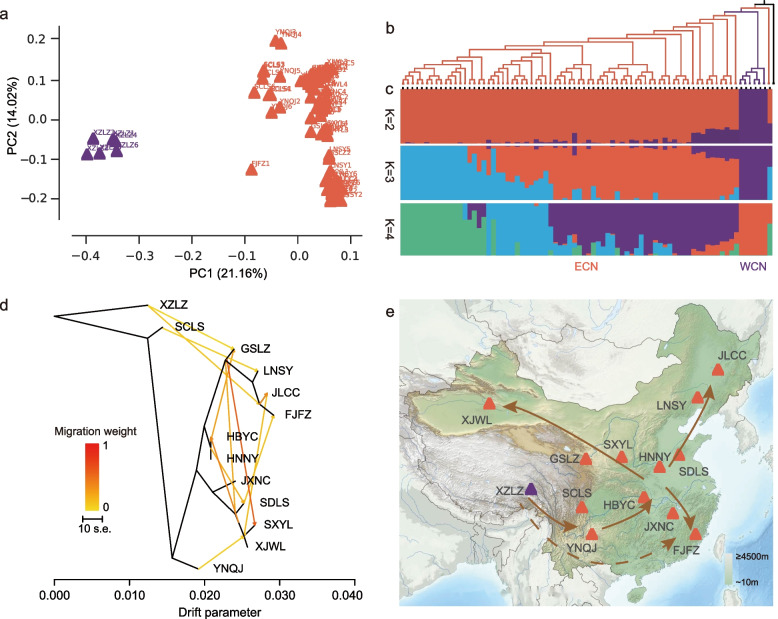
Population structure and geographic expansion of the *Harmonia axyridis* populations within China. **a** A biplot of the first two components obtained from the principal component analysis of geographic sample populations clustered into two major groups (super-populations) represented by colors of purple for the west China (WCN) and red for the east China (ECN). **b** A phylogenetic tree of *H. axyridis* populations reconstructed with *Agrilus planipennis* as the outgroup and with the WCN group as the closest to it. **c** Population structure of sample populations (*K* = 2, 3, and 4) matching the phylogenetic tree with colored (same as above) triangles representing respective components in each population from the two major groups. **d** Splits and migrations among sample populations with shades of color representing the weight of migration. **e** A schematic diagram showing putative origin and dispersal (with arrows representing the direction) of the *H. axyridis* populations represented by triangles with colors of purple for the west China (WCN) and red for the east China (ECN) super-populations

The PCA of the 7824 polymorphic SNPs within *H. axyridis* populations sampled from its native range China (ECN and WCN super-populations) indicated that the first two principal components accounted for 35.18% of the total variance, with PC1 reflecting the separation of XZLZ in Tibet from the other Chinese populations (Fig. 3a). This pattern was in line with the phylogenetic relationship and population structure estimates (at *K* = 2) (Fig. 3b, c; Additional file 2: Fig. S1b). Two populations (YNQJ and SCLS) in southwestern China were admixed with XZLZ as well as with other Chinese populations (Fig. 3c). At *K* = 3, populations in northeastern China (e.g., JLCC and LNSY populations) were identified as their own structured cluster. At *K* = 4, the XJWL, GSLZ, and SXYL populations in northwestern China could be readily distinguished (Fig. 3c). Moreover, the analysis of splits and migration rates among geographical populations in China showed that *H. axyridis* originated in southwestern China and spread from there to the other regions (Fig. 3d, e).

### Patterns of contemporary and historical global migration

**Fig. 4:**
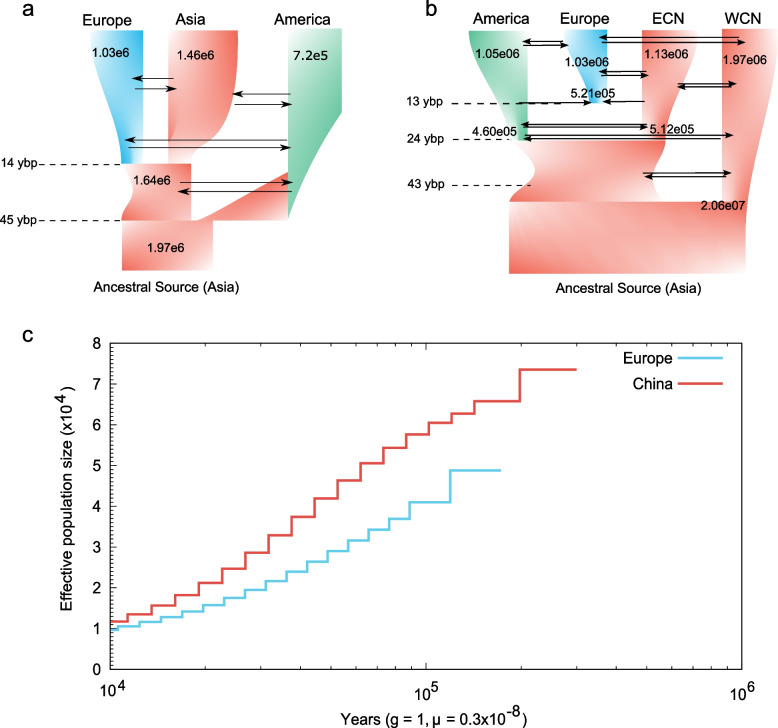
Demographic history and genome-wide patterns of differentiation of major groups (super-populations) of *Harmonia axyridis*. **a** The most likely evolutionary demographic history of global invasion of *H. axyridis* as estimated from FSC26 analyses under the 3-population model, suggesting a recent invasion (~ 14 ybp) of the Europe super-population from an Asian source, with significant yet low continued gene flow from the America super-population. Additionally, the estimated population size of the America super-population is considerably smaller compared to the European and two Asian super-populations, the detailed information was shown in additional file 1: Table S5-S7. **b** The most likely evolutionary demographic history of global invasion of *H. axyridis* as estimated from FSC26 analyses under the 4-population model, indicating recent (~ 13 ybp) hybrid origins of the European super-population as a result of admixture between the Eastern China and the America super-populations, which themselves diverged from their common ancestor ~ 24 ybp. The Western China and the Eastern China super-populations were estimated to have diverged around 43 ybp. The America, Europe, and Eastern China super-populations have undergone significant increase in effective population sizes after recovery from bottlenecks, while the Western China has decreased in size since divergence, the detailed information was shown in additional file 2: Table S8-S9. **c** Historical variation in effective population sizes estimated from the whole genome resequencing data sourced from Chen et al., 2021 (native population from China), and Boyes et al., 2021 (invasive, non-native population from the UK, Europe). All estimates were scaled by a generation time of 1 generation per year, and a mutation rate of 3.5e − 9 per site per generation

Reconstruction of demographic history using FSC26 indicated that the best fitted 3-population model suggested asymmetric migration under topology t1, with a most recent divergence of the Europe super-population from the Asia (~ 14 ybp) and an earlier divergence of the America from the common ancestral source population in Asia (~ 45 ybp, Fig. 4a; Additional file 1: Table S5, S6). The America super-population had a lower effective estimated population size than the Asia and Europe super-populations, suggesting a series of bottleneck events leading to reduced genomic diversity in the non-native populations. The estimation of contemporary migration under the island model using BA3-SNPs indicated that a majority of recent migrations occurred within sampled main ranges (Americas, Europe, and China) and not between them (Additional file 1: Table S7).

The estimation of evolutionary demographic history under the 4-population topology with recent hybrid origins of the Europe super-population provided strong support for the model of isolation with migration and exponential change in population sizes post divergence (Fig. 4b; Additional file 1: Table S8, S9). The 4-population model estimates were also largely in line with those under the 3-population model, in that (a) the America and Europe super-populations are estimated to be smaller than the east and west China super-populations, (b) contemporary migration rates between the four super-populations were low (congruent with the BA3-SNPs estimates), (c) the hybrid origins of the Europe super-population were estimated to be recent, of the order of 13 ybp, while the divergence of the America super-population from the east China super-population was estimated to have occurred around 24 ybp, and (d) the divergence between the east and west China super-populations was estimated at 43 ybp. Additionally, all non-native (introduced) populations were determined to have had significant population size expansions after the initial introduction bottleneck, whereas the native, west China super-population was noted to have undergone a ten-fold bottleneck from its ancestral size.

### Historical variation in effective population sizes

Our PSMC analyses of historical variation in effective population sizes using high quality *H. axyridis* whole genomes, one being representative of its native range in China [43] and another its non-native range in Europe [44], indicated (1) signatures of a large progressive, historical bottleneck in the common ancestors of both populations, (2) commensurating in similar bottlenecked populations around 10,000 ybp, (3) smaller effective sizes of the non-native population through time (~ 50,000; 100,000 ybp) compared to the native population (~ 75,000; 100,000 ybp), and (4) more “recent” coalescence of the European *H. axyridis* genome compared to the Chinese *H. axyridis* genome (Fig. 4c).

### Analyses of signatures of natural selection

Analyses of outliers using (a) genome-wide distribution of Weir & Cockerham’s *F*_*ST*_ across four global superpopulations, (b) estimates of *F*_*ST*_ outliers by Arlequin, and (c) OutFLANK analyses across all sample populations indicated that a single outlier locus (scaffold 1381, site 494,868) was significantly differentiated across all sample populations and was mapped back to the Histone-lysine-N-methyltransferase SETMAR locus in the *H. axyridis* genome (Additional file 2: Fig. S2a, b).

Several of the top ten outlier loci identified by our genome-wide scan of *F*_*ST*_ and Arlequin also mapped back to the neighborhood of the highly variable *pannier* locus (Additional file 2: Fig. S2c), with a couple of loci homologous to the carbamoyl-phosphate synthetase gene (CADII), which is involved in hemolymph production in coccinellid beetles. Other loci that were identified to be among the top 95% percentile of outliers by Arlequin included the procathepsin L-like locus on the X-chromosome, ATP-dependent RNA helicase spindle-E, and piggyBac transposable-element derived protein (Table 1). We acknowledge that outliers on the X-chromosome could potentially be false-positives, owing to us not controlling for sex of genotyped individuals.

**Table 1:** Top ten Weir and Cockerham’s *F*_st_ outlier loci identified across all sampled populations using Arlequin, and their homology to known functional genes from GenBank

Chromosome	SNP position	Locus	Obs. Het. BP	Obs *F*_*ST*_	*P*-value	Location	Protein
Original_scaffold_1348	5,014,802	6747	0.202924	0.882225	0.00E + 00	Downstream of LOC123671460	Probable ATP-dependent RNA helicase spindle-E
FragScaff_scaffold_198	4,590,085	4739	0.211016	0.875361	1.05E − 299	Unknown	Unknown
Original_scaffold_540	399,348	6068	0.190675	0.854398	1.05E − 273	Downstream of LOC123686084	*piggyBac* transposable element-derived protein 3-like
FragScaff_scaffold_198	4,755,908	4743	0.214471	0.849187	3.44E − 267	Unknown	Unknown
FragScaff_scaffold_198	5,597,777	4767	0.165288	0.775155	1.02E − 216	LOC123685704	Procathepsin L-like (LOC123685704), transcript variant X2, mRNA
Original_scaffold_1348	1,942,409	6691	0.23695	0.79731	1.57E − 208	Downstream of LOC123671460	Probable ATP-dependent RNA helicase spindle-E
Original_scaffold_1348	3,619,473	6711	0.226716	0.773365	8.86E − 184	Downstream of LOC123671460	Probable ATP-dependent RNA helicase spindle-E
Original_scaffold_1348	4,905,076	6744	0.221071	0.76982	3.08E − 180	No match	No match
FragScaff_scaffold_60	2,102,089	1125	0.239309	0.758214	3.51E − 169	Upstream of *pnr* locus	Uncharacterized protein LOC123676881

### Life history trait variation among H. axyridis haplotypes

**Fig. 5:**
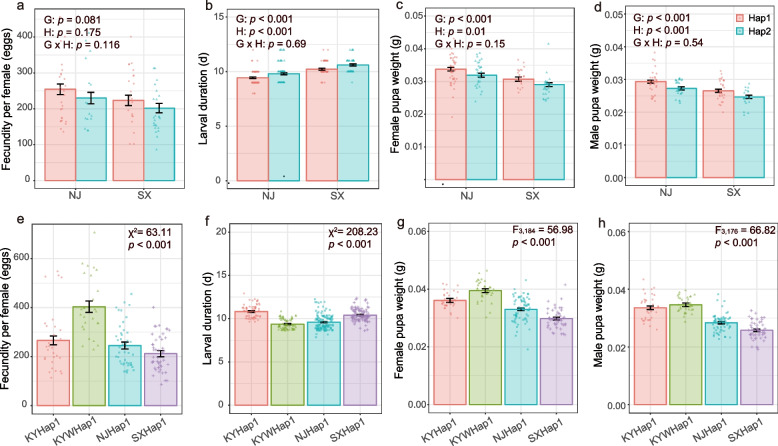
Life history traits between *Harmonia axyridis* haplotypes in native and non-native ranges. **a**–**d** Life history trait distribution of the two dominant haplotypes Hap-1 and Hap-2 of *H. axyridis* in its native range. Legend: G = geographic site, H = haplotype, G × H = interaction between geographic site and haplotype. **e**–**h** Life history traits of Hap-1 *H. axyridis* in its native (NJHap-1 and SXHap-1, China) and invaded (KYHap-1 and KYWHap-1, USA) ranges

Under controlled laboratory conditions, we determined if Hap1, the only mtDNA haplotype we detected in all invaded regions, had any phenotypic traits that might be associated with the invasion success of *H. axyridis*. The fecundity between Hap1 and Hap2 *H. axyridis* from China was not significantly different (*P* = 0.11) (Fig. 5a), while the Hap1 completed larval development by half a day faster (*P* = 0.0002) (Fig. 5b) and formed heavier pupae by 3.9 mg for male (*P* = 0.01) and 4.1 mg for female (*P* = 0.001) than the Hap2 (Fig. 5c, d). *H. axyridis* Hap1 ladybirds from Princeton, KY, USA, had significantly higher fecundity than that from Nanjing (*P* < 0.001) or Yangling (*P* < 0.001) in China, while the Hap1 from Lexington, KY, USA, was not different from that from either Nanjing (*P* = 0.79) or Yangling (*P* = 0.06) (Fig. 5e, f). Male and female *H. axyridis* Hap1 pupae from the USA were heavier (*P* < 0.001) than their counterparts from China (Fig. 5g, h).

## Discussion

Our comprehensive analyses of global patterns of* H*. *axyridis* invasion (1) established a timeline and sequence of invasion events indicating pervasive spread of a single mitochondrial haplotype (Hap1) from China; (2) characterized genome-wide signatures of selection in non-native and native populations, particularly at loci linked to the color morph *pannier* locus; (3) discovered in laboratory experiments made in both China and USA that *H*. *axyridis* mtDNA haplotype (Hap1), dominant in the putative original populations in the native range and extant only in the non-native range, had higher lifetime fecundity and improved performance in other life history traits as compared to another main haplotype (Hap2) derived from Hap1 in the native range; (4) delineated the geographical origin of *H*. *axyridis* and dispersal routes across mainland China in its native range; and (5) established historical variation in demographic history of establishment and spread of *H. axyridis*.

*Harmonia axyridis* is generally known to be native to continental, temperate and subtropical parts of east and central Asia [45, 46], yet its geographic history within this region remains largely unknown. Our analyses of divergence and migration rates among Chinese populations suggest that it might originate in southwestern China, thence spread eastward and then both north- and westward across China. The southwest part of China is widely acclaimed for its Hengduan Mountains region (HDM) as one of the earth’s 34 biodiversity hotspots [47]. The HDM is part of the Tibeto-Himalayan region which includes a series of parallel, mostly north to south oriented high mountains separated by deep valleys with elevations ranging from 1000 m on valley floors to > 6000 m on mountain peaks [48, 49]. The HDM is not only a natural historical “museum” that has preserved plant diversity since the Cenozoic era but also a “cradle” where many new species were formed [47]. Such highly diverse habitats and flora are assumed to be coevolved with a rich phytophagous insect fauna. Rich prey species concurred with diverse habitats may facilitate speciation in predatory lady beetles in this region. As supporting evidence, the genus *Harmonia* is most speciose in and most of its species are endemic to the southwestern part of China [50]. We thus assume that *H. axyridis* emerged in the HDM region or nearby and spread from there to other regions in its current native range.

Previous studies that analyzed patterns of microsatellite variation and estimated migratory history suggest the establishment of North American populations from Eastern and Western China in 1988–1991, followed by subsequent importation of the American populations into South America and Africa, and a separate admixture event between the Eastern Chinese populations and American populations to establish the European populations [42]. Our study using genome-wide SNP variation describes the evolutionary history of global populations, suggesting a recent divergence and putative hybrid admixed origins of the modern European populations, composed of genomes originating from eastern North American and eastern Chinese populations, with little continued contemporary gene flow between them and with significant bottlenecks in all invaded populations. It needs to be noted that a few populations from central Europe were clustered with Eastern and Western China groups. Our supposition is that the ladybird might have dispersed from the originally arrival places (France and Belgium) where the introduction of biological control populations was first made. Another supposition is that there was a gene exchange between ladybird populations from which our samples were collected and those introduced populations. Comparative analyses of historical effective population sizes using two whole genomes [43, 44] also indicate a large historical bottleneck event in the pre-establishment past of both native and non-native *H. axyridis*, along the timelines of the last glacial maximum (100,000 ybp–10,000 ybp). Similar declines with glacial recession have been reported in other insects, such as the scarce heath, *Coenonympha hero* [51], and the darkling beetle, *Nyctelia confusa* [52]. However, we found that the heterozygosity values (nuclear) were higher in non-native than in native populations. A similar pattern was identified in a genetic analysis using microsatellite markers, which demonstrated a trend toward an increase in heterozygosity in some of invasive *H. axyridis* populations, closest to the North European core of invasion [53]. Similarly, we discovered a high *mtCOI* haplotype diversity based on a single population collected from South America with relatively small sample size, where *H. axyridis* was introduced ca. 20 year ago from North America. Perhaps, the single population is insufficient to represent the invasive population in the South America and/or it is likely that there are many invasive populations with different origins in South America.

The *F*_*ST*_ outlier loci indicated by several lines of evidence (OutFLANK and genome-wide windowed Weir and Cockerham’s* F*_*ST*_ estimation) showed that the histone-lysine-N-methyltransferase (SETMAR) locus has putatively undergone selection pressure across both the native and the non-native regions. This locus, which has been implicated in dsDNA break repair and control of replication, is involved in reproductive regulation, epigenetic maternal imprinting, and polymorphism regulation in other insects, such as the rice brown planthopper *Nilaparvata lugens* [54] and the leafcutter ant *Acromyrmex echiniator* [55]. Interestingly, this locus is found to be associated with range expansion and local adaptation across a variety of novel environments in the damselfly, *Ischnura elegans* [56]. Another DNA methyltransferase (DMAP1) complex is also directly associated with female fecundity in *H*. *axyridis* [57], wherein gene silencing was strongly implicated in ovarian degeneration and reduced fecundity.

However, an important discovery revealed by our analyses of homology of the scaffolds containing these significantly differentiated variants as aligned with the annotated contigs clearly indicates genomic synteny with intronic regions linked and in the immediate vicinity of the *pannier* (*pnr*) locus [58]. This locus has been previously reported to be closely associated with color morph variation in *H. axyridis* [58, 59]. This suggests that the non-native and native *H*. *axyridis* populations are undergoing strong selection pressure in the intronic regions linked to the *pnr* locus, with nearby allelic variants hitchhiking to fixation within the three global super-populations. Yet, recombination between *pannier* alleles may be reduced by a highly divergent sequence (~ 170 kb) in the *cis*-regulatory regions of *pannier*, leading to highly variable discrete color forms in natural populations [58]. Interestingly, a previous study also suggests the exclusive presence of non-melanic morphs of *H. axyridis* in its invaded American range [60]. This implies the linked selection effects in non-native populations leading to fixation of genomic loci around the *pnr* locus. Also, a previous study have shown that Other loci that were putative *F*_*ST*_ outliers included a locus linked to the procathepsin L-like protein, which is a digestive proteinase in Coleoptera [61] and the *piggyBac* locus which shows incredible diversity among insect orders [62]. We would however encourage additional whole genomic analyses to test these linked selection effects further, in that *F*_*ST*_ outlier analyses such as those we utilized are potentially confounded by demography and selection [63–66].

We found the sole occurrence of the mitochondrial haplotype Hap1 across all globally non-native *H*. *axyridis* populations, while both Hap1 and hap2 were dominant in native populations from China. Our subsequent laboratory observations in both USA and China showed that Hap1 ladybirds were more fecund, with a higher pupal mass and a shorter larval duration than Hap2 ones. Studies of insects such as fruit flies [67, 68] and planthoppers [69] suggest that mitochondrial DNA variation can be associated with life history traits. So, we assume that Hap1 of *H*. *axyridis* may be more invasive than the Hap2, leading to its invasion success in North America by life history advantages. Yet, an alternative possibility is that Hap2 has been eliminated by strong negative selection in the non-native range due to lacking the standing genetic variation for adaptive potential in novel environments. Regardless, our genomic outlier analyses indicate the presence of selection pressure and linked selection across potential causal loci to differential fitness phenotypes, such as in color polymorphism and reproductive traits. The absence of Hap2 in the non-native range may be also potentially due to serial bottleneck effects and stochasticity in its establishment. Additionally, extra caution must be exercised about the life history difference performed by *H*. *axyridis* ladybirds between Hap1 and Hap2 due to uncontrollable differences in some factors (e.g., experimental conditions, diets) between the two observation sites.

As discussed in a recent review, research attention is urgently needed to focus on the characteristics of and interactions among *H. axyridis* and co-existent species of ladybirds in their native Asian habitats [30]. Our current study has documented significant genetic and phenotypic differences among native and non-native populations of *H. axyridis*. Non-native populations of *H. axyridis* are currently spreading from Europe into western Asia [70]. Therefore, hybridization between native and non-native populations will likely occur, but may be complex due to a number of differences between the populations [30]. Based on our comparisons of the life history traits of native and non-native populations of *H. axyridis*, field research is required to examine the potential effects of the non-native population on the biodiversity of ladybirds in Asia and their role in the biological control of agricultural pests.

## Conclusions

Our analyses of genome-wide nuclear population structure of *H. axyridis* samples from across its native and non-native ranges indicated that an eastern Chinese population could be the source of all non-native populations, revealed several putatively adaptive candidate genomic loci, and reconstructed a geographic range evolution within its native range in China. These findings provide new insights into its invasion processes into other major continents from its native Asian range. Our comparative observations of life history traits of the two main mitochondrial haplotypes in laboratories of both China and North America tentatively suggests that *H. axyridis* invasiveness may differ between haplotypes.

## Methods

### Sample collection

For *mtCOI* sequencing, 1025 *H. axyridis* adults were collected from 29 geographical localities across mainland China, from nine sites across North and South America and from four across Europe (Additional file 1: Table S10). Adults were collected using sweep-nets from June through October 2017–2019 mainly from various habitats including grassland, vegetable fields, garden shrubs, orchards, and soybean fields. All samples were stored in vials containing 95% ethanol at – 20 °C/ − 80 °C prior to DNA extraction.

Our previous analyses conclude that six ladybird individuals for each locality can yield a reliable estimate of population genomic parameters such as genetic diversity and estimates of population genetic differentiation with IIB-digest restriction site-associated DNA (2b-RAD) sequencing and library construction [71]. So, we applied a sample size of *n* = 6 individuals (regardless of their morph variation and habitats) taken at random from the collection with > 6 individuals from each locality at 26 localities, while applying a sample of *n* = 5 individuals from a locality with < 6 ones for the other 3 localities (Additional file 1: Table S10). These locations were chosen to maximize the coverage of *H. axyridis* populations across its native and non-native ranges, at altitudes varying from a few hundreds to ca. 4000 m in Tibet in China.

The colonies of *H. axyridis mtCOI* haplotype for life history observations were established in insectaries in both China and USA. In 2018, two colonies of both Hap1 and Hap2 were established at an insectary at Nanjing Agricultural University, Weigang, Nanjing, from collections in two localities: one in Yangling District (latitude 34° 27′, longitude 108.08°), Xi’an city in the Shanxi province, western China; another one in Pukou district (latitude 32° 05′, longitude 118.67°), of Nanjing city in Jiangsu province, eastern China. In 2020, two Hap1 colonies were established at an insectary at University of Kentucky, Lexington, from collections in Lexington and Princeton (separated by 338 km), respectively, in Kentucky, USA. About 50 pairs of adults from each locality were individually transferred to Petri dishes (9 cm in diameter) for mating and oviposition. After laying eggs, these adults were reserved for subsequent *mtCOI* haplotype identification; a random sample of the 1st instar larvae hatched from those eggs were also taken for *mtCOI* haplotype identification. Ladybird larvae were subsequently grouped by haplotype and reared in cages at the temperature of 25 ± 1 °C, RH 65 ± 5%, and 16:8 light to dark photoperiod. The haplotype cohorts were maintained for three generations prior to the life history observation, with provision of the bean aphid *Megoura japonica* (Matsumura) in China and the pea aphid *Acyrthosiphon pisum* (Harris) in the USA, that were reared on the same host plant *Vicia faba*.

### DNA extraction

A total of 1025 unsexed adults from China and Europe were individually subjected to total genomic DNA extraction using the EasyPure Genomic DNA Kit (Transgen Biotech, China) by following the manufacturer’s protocol, while those from the non-native locations in the Americas were processed using the Dneasy Blood & Tissue Kit (Qiagen, Germany). The extracted genomic DNA from individual beetles was stored at – 20 °C until *mtCOI* fragment amplification and haplotype identification as well as 2b-RAD sequencing.

### Mitochondrial haplotype identification

The *mtCOI* gene was amplified using the LCO1490 (5′-GGTCAACAAATCATAAA GATATTGG-3′) and HCO2198 (5′-TAAACTTCAGGGTGACCAAAAAATCA-3′) primers [72]. PCRs were performed in 25 μL solutions containing 22 μL 1.1 × buffer (Tsingke, Biotech), 1 μL (10 μM) of each primer, and 1 μL of template DNA using the following thermocycling program: initial denaturation at 98 °C for 2 min, followed by 35 cycles of denaturation at 98 °C for 10 s, primer annealing at 50 °C for 10 s, and extension at 72 °C for 1 min, and a final extension at 72 °C for 2 min. Amplified products from samples from China, Europe, and South America were sequenced bi-directionally using an ABI 3730 DNA Analyzer at Genscript Biotech (Nanjing, China), and those from North America were sequenced using Sanger sequencing method at Functional Biosciences, Inc. (Wisconsin, USA).

All *mtCOI* sequences were aligned using ClustalW in MEGA 6.0 [73]. Gaps and indels that were identified across all alignments were manually checked and 620 bp of *mtCOI* sequence was obtained from each sample. Assignment and measurements of mitochondrial haplotypes, including haplotype diversity (*H*_*d*_), nucleotide diversity (*π*), and the average number of nucleotide differences (*K*), were performed using DnaSP 5.0 [74]. The confirmation of haplotypes was done following the technique devised by De Barro and Ahmed [75]. The neighbor joining phylogenetic tree using Kimura-2-Parameter algorithm with bootstrap values indicated on the branch, and the *mtCOI* sequence (NCBI Accession: KU918664) of the variegated ladybird *Hippodamia variegata* (Goeze) [previously *Adonia variegata* (Goeze)] as an outgroup. Pairwise differentiation (*F*_*ST*_) between sampling locations was estimated with the Arlequin v.3.5.1.2 program [76] using the Tamura–Nei estimator [77]. The haplotype network of the *mtCOI* genes was inferred using the median-joining algorithm in the software Network v.4.6.1.0 (Fluxus Technology Ltd., England) [78].

### Life history observations

Observations of life history traits were made in the laboratory on two mitochondrial haplotypes, Hap1 and Hap2, of *H. axyridis* in both China (Hap1 and Hap2, at Nanjing Agricultural University, Nanjing, Jiangsu province) and USA (Hap1, at University of Kentucky, Lexington, KY). To control for the potential effects of confounding factors, the environments for rearing *H. axyridis* cohorts in the two insectaries were identical (temperature 25 ± 1 °C, RH 65 ± 5%, and photoperiod 16:8 light to dark). In addition, due to a lack of same prey species available for rearing the ladybird in different insectaries, two different but related aphid species were employed as diets: *M. japonica* in China and *A. pisum* in the USA. These two aphid species are in the same tribe of aphids (Macrosiphini), both feed on plants in the family Fabaceae, and both aphid species were reared on the same host plant species the broad bean *V. faba* for the experiments in China and the USA. Moreover, all the observations and insect-rearing procedures in both China and USA were conducted by the same individual (the first author H. L.). Neonate ladybird larvae were individually transferred into vials where aphids were supplied ad libitum as prey. They were reared at the temperature of 25 ± 1 °C, RH 65 ± 5%, and 16:8 light to dark photoperiod. Larval development was observed daily until pupation, and pupae were individually weighed (Mettler Toledo, model AL204-IC, to an accuracy of 0.01 mg). Emerged adults were paired in Petri dishes where aphids were provided ad libitum as food. After oviposition started, eggs were counted each day for 20 days, during which female ladybirds lay most of their eggs; thus, the total number of eggs laid during this period was used as a measure of fecundity.

A generalized linear model was fit to fecundity and larval duration (count variable), using quasi-Poisson error distribution when data were overdispersed [79], and a general linear model was fit to pupal body mass (continuous variable), as a function of independent variables of the haplotype (Hap1 and Hap2) and the site (China and USA). All models were visually checked for assumptions of normal distribution of residuals and homoscedasticity. After detecting a significant effect, the Tukey comparison procedure was used to make pairwise contrasts of least square means estimated in the model using the R package *emmeans* [80]. Statistical significance was determined at α = 0.05 (two-tailed). All statistical data analyses were done using the R statistical software version 3.3.1 [81].

### 2b-RAD library construction, sequencing, and SNP calling

Approximately 200 ng of whole genomic DNA from 159 representative individuals were digested using Type IIB BsaXI restriction endonuclease, followed by the addition of five unique adapter sequences with T4 DNA ligases. The adapter ligated restriction products were PCR amplified, barcoded, and pooled prior to paired-end sequencing on an Illumina NovaSeq at OE Biotech (Qingdao, China) [82]. Raw reads were filtered by quality (PHRED Q score >  = 30 for at least 75% of each the length of each read, < 8% missing data or N bases, and the reads that did not contain BsaXI restriction sites), and all paired-end raw reads were merged using the Pear (v.0.9.6) program and assembled using the *ustacks* (v.1.3.4) program with the *H. axyridis* reference genome (http://v2.insect-genome.com/Organism/418) [43] using the SOAP aligner (-r0 –M4 –v2) [83, 84], allowing for a maximum mismatch of 2 nucleotide bases, and filtering for unique and optimal autosomal and X chromosome alignments. Picard Tools (http://broadinstitute.github.io/picard/; v1.118) was used to estimate, sort, remove PCR duplicates, and build BAM indices. Ambiguously mapped reads were removed from further analyses. Alignment files were then converted to BAM files using SAMtools v0.1.18 [85]. Variant calling was performed for all samples using the Genome Analysis Toolkit (GATK, version 3.6–0-g89b7209) by filtering out sites based on the following criteria [86]: (a) minor allele frequency (MAF) of < 0.05, (b) more than 2 alleles, and (c) > 20% of missing data. This resulted in a total of 7824 SNP sites across all samples. The SnpEff (v 5.0) program was used to annotate all SNPs against the annotated reference genome GFF3 file [87]. The RAD-seq data analysis procedure are shown in additional file 2: Fig. S3.

### Genetic diversity and population structure analysis

The phylogenetic tree of all *H. axyridis* geographical populations was constructed based on the neighbor-joining algorithm with 1000 bootstrap replicates using the Treebest (v1.9.2) program [88]. A principal component analysis (PCA) was also conducted to evaluate the genetic structure of the populations using Plink v1.90) [89]. Additionally, population structure and admixture proportions were estimated using ADMIXTURE v.1.30 with *K* = 2–15 subpopulation clusters [90], terminating at convergence at an objective function delta of < 0.0001, and using the Quasi-Newton acceleration with 3 secant conditions. The best fitting number of superpopulations was determined using the *K* value with the lowest cross-validation error. Heterozygosity (*H*_*o*_), nucleotide diversity (*π*), genetic differentiation (*F*st) across the four global superpopulations (ECN, WCN, AME, and EUR) as estimated by our ADMIXTURE analyses (at *K* = 3) were calculated using VCFtools (v0.1.16) in 10-kb nonoverlapping sliding windows along each chromosome [91]. We utilized a conservative 10-kb window, based on the Coleopteran recombination rate of ~ 3 cM/Mb [92], based on a ~ 400 Mb genome, which indicates approximately one recombination event every 13-kb of the genome.

For the Chinese populations, we conducted the population genetic analysis including PCA, phylogenetic tree, and population structure analysis using identical SNP dataset (7824 SNPs). Notably, to further infer population-level splits and mixtures for *H. axyridis* populations from China, we filtered SNPs obtained by GATK with the following criteria: the max missing rate was 90%, collating a total of 4429 high-quality genotyped SNPs. Then, we used the Treemix 1.13 to investigate the admixture between populations, with migration edges ranging from 1 to 20 [93].

### Estimation of contemporary and historical migration events

Prior to population genomic analyses, further filtering was applied using BCFtools v.1.11 to remove (a) the sites that were not in Hardy–Weinberg equilibrium (*P* < 10^−4^) for analyses of population structure, genetic diversity, and estimates of contemporary and historic migration and (b) the sites in linkage disequilibrium (*R*^2^ > 0.6 in 1000 sites) that were filtered for *F*_ST_ outlier analyses. All population genomics analyses were performed with the PPP pipeline [94] using the BCFtools v.1.11 (https://samtools.github.io/bcftools/bcftools.html).

Estimates of contemporary migration rates were estimated using the Bayesian MCMC method of the BayesAss3-SNPs (BA3-SNPs) [95, 96] by using a burn-in of 10^7^ steps, followed by sampling 5 × 10^7^ steps (sampling every 1000th iteration). Convergence and mixing of the MCMC runs were assessed using the Tracer v.1.7.1 program [97], and posterior density estimates of contemporary pairwise migration rates and their 95% confidence intervals were obtained.

### Estimation of evolutionary demographic history

Demographic modeling of the coalescent evolutionary history was performed using the Fastsimcoal26 package (FSC26) [98]. We combined individual beetles based on their sampling locations into three super-populations; this was done in congruence with the most optimal population structure estimated by ADMIXTURE, at *K* = 2, with the European population comprising of admixed Asian-American beetles: Asia, Europe, and America, and generated two-dimensional folded (minor allele) site frequency spectra (SFS) from the complete 2b-RAD dataset. With three super-populations, we considered four possible population tree topologies, shown in the NEWICK format: (1) t1: (((Asia, Europe), MRCA1), America), MRCA2; (2) t2: (((Asia, America), MRCA1), Europe), MRCA2; (3) t3: (((Europe, America), MRCA1), Asia), MRCA2; and (4) t4: (Asia, Europe, America), MRCA. We considered four possible models under each topology: (1) no migration between sampled populations (isolation with no migration), (2) asymmetric migration between sampled populations (isolation with migration, IM), (3) asymmetric migration between sampled populations (IM) with exponential growth of each population since divergence, and (4) no migration between sampled populations with exponential growth of each population since divergence (Additional file 2: Fig. S4). Additionally, based on the most likely topology and demographic history estimates from the three super-population FSC26 runs, we constructed four more scenarios: (1) no migration between populations, (2) asymmetric migration between populations, (3) no migration with exponential growth, and (4) asymmetric migration between populations and exponential growth of a four-population topology (Additional file 2: Fig. S5), such that the Asian group was split into ECN and WCN groups, with the European group formed as a result of hybridization between the American and ECN groups, which fits the *K* = 3 super-population structure estimates from the ADMIXTURE.

Subsequently, we then performed a series of analyses in the FSC26 to obtain estimates of demographic history: (1) 100 replicate runs of 200,000 coalescent simulations in each cycle, with 40 optimization cycles, discarding monomorphic sites, and sites with < 10 SNP’s in the SFS for estimating demographic parameters; (2) under each such model (4 three population topologies × 4 models × 100 runs each = 1600 total runs, 1 four-population topology × 4 models × 100 runs each = 400 total runs), computing AIC from the “best” likelihood scenario; (3) picking the “best” most likely model based on the AIC estimate, and simulating a total of 100 parametric bootstrap datasets of 200,000 non-recombining 100 bp DNA segments each; and (4) re-running estimation of demographic parameters, similar to step (1) from all 100 of these parametric bootstrap datasets to obtain confidence intervals around the estimates. All these estimates were scaled using an estimated mutation rate of 3.5 × 10^−9^ per site per generation for *Drosophila melanogaster* [99]. Goodness of fit of the observed 2D site frequency spectra to that expected under the best fitting three-population and four-population demographic models were visually assessed to ensure congruence (Additional file 2: Fig. S6, S7).

### Analyses of signatures of natural selection

We used the outlier detection *F*-statistics implemented in Arlequin v.3.5.2.2 [76] by grouping individuals according to their global superpopulations (AME, EUR, WCN and ECN), and performing 20,000 simulations of 100 demes per group, with 10 groups of 100 demes to establish the neutral expectations of *F*-statistics under a hierarchical island model. Subsequently, *F*_*ST*_ outlier loci in our empirical distribution of 7824 SNPs were identified as loci in the top 1 percentile tail of the estimated distribution after the FDR (Benjamini and Hochberg) correction for multiple tests. Additionally, after thinning the dataset for LD using BCFtools, we performed analyses of *F*_ST_ outlier loci using OutFLANK [100]. Weir and Cockerham *F*_ST_ estimates were computed across all sampled populations (i.e., without any grouping into global superpopulations), and uncorrected *F*_ST_ estimates were used to ensure that loci were not deviating from the corrected *F*_ST_ estimates. The OutFLANK function was then used to compute deviating loci from the expected neutral *F*_ST_ distribution, and outliers were estimated at a *P*-value cutoff of 0.05 using the FDR (Benjamini and Hochberg) correction for multiple testing. Outlier loci were mapped back to the raw reads from our 2b-RAD sequencing, and NCBI BLAST-n was used with publicly available Coleoptera: Coccinellidae genomes to obtain highly similar “hits.”

### Analyses of historical effective population size variation in native versus non-native ranges

We inferred historical variation in effective population sizes of *H. axyridis* in its native range China versus in its non-native range Europe using two published resequenced whole genomes (Accession ID: SRR1348220, Beijing Academy of Agriculture and Forestry Sciences; Accession ID: ERR6054990, Wytham Woods, Oxfordshire, UK) [43, 44]. Briefly, we utilized the consensus reference genome to construct whole genome alignments using Bwa-mem2 v.2.0 [43, 101] and then sorted and constructed a consensus sequence using Samtools v.1.16.1 [88]. The consensus sequence was then converted into the PSMC FASTA format using the fq2psmcfa tool, filtering for sites with a PHRED Q score greater than 30. We then utilized PSMC v.0.6.5-r67 [102] with a generation time of 1 year, and a per locus mutation rate of 3.5 × 10^−9^ per site per generation, an upper limit of 5 for TMRCA, using 34 free atomic intervals [103]. Results of historical variation in effective population sizes were then visualized and compared between the native (China) and non-native (Europe) genomes using the psmc_plot tool.

## Supplementary Information

**Additional file 1:** **Table S1.** Polymorphic nucleotide position of *mtCOI* gene defining the 22 haplotypes identified in native and non-native* Harmonia axyridis* populations. **Table S2.** Genetic diversity indices of native and non-native *Harmonia axyridis* populations based on 620-bp *mtCOI* fragment. **Table S3.** Pairwise *F*_*ST*_and migration rateof *Harmonia axyridis *populations from native and non-native regions. **Table S4.** Individual estimates the number of observed and expected homozygote sites, observed heterozygosity, and a method of moments estimate of the individual inbreeding coefficient. **Table S5.** Estimates of Maximum likelihood and Akaike’s information criterionacross different 3-population evolutionary demographic models. **Table S6.** Parameter estimates of genetic diversity, migration rate, divergence time, and population size change under the most likely 3-population topologyunder the IM model with population size changes across three major groups of *Harmonia axyridis* populations worldwide. **Table S7.** Estimates of contemporary migration between three major groups of *Harmonia axyridis* populations as estimated withBA3-SNPs. **Table S8.** Estimates of Maximum likelihood and Akaike’s information criterionacross different 4-population evolutionary demographic models. **Table S9.** Parameter estimates of genetic diversity, migration rate, divergence time, and population size change under the most likely 4-population topologyunder the IM model with population size changes across four major groups of *Harmonia axyridis* populations worldwide. **Table S10.** Collection information of *Harmonia axyridis* samples and their haplotypes from native and non-native ranges.

**Additional file 2:** **Fig. S1.** Cross validation errors computed with ADMIXTURE v.1.3.0 by varying the number of superpopulations between K = 1 and 15. **Fig. S2.** Differentiation measured as* F*_*st*_versus all chromosomal locations from OutFLANK analyses. **Fig. S3.** The workflow analysis for population genomics of the harlequin ladybird, *Harmonia axyridis* in the present study. **Fig. S4.** Demographic models of global invasion history of *H. axyridis* from an ancestral Asian source population tested in FSC26 between 3 superpopulations. **Fig. S5.** Demographic models of global invasion history of* H. axyridis *from an ancestral Eastern China source population tested in FSC26 between 4 superpopulations. **Fig. S6.** Goodness of fit estimates of the observed and estimated two dimensional site frequency spectra from our best fitting four-population model comprising asymmetric migration and exponential population size change. **Fig. S7.** Goodness of fit estimates of the observed and estimated two dimensional site frequency spectra from our best fitting three-population modelcomprising asymmetric migration and exponential population size change.

## Data Availability

All data generated or analyzed during this study are included in this published article, its supplementary information files, and publicly available repositories. All raw data of 2b-RAD sequencing generated during this study have been deposited at the National Center for Biotechnology Information (NCBI) under the accession numbers of PRJNA850112 [104]. All sequences of 22 *mtCOI* haplotypes have been deposited at GenBank with the accession number: MT826903-MT826924. All scripts, files, and code utilized in the population genomic analyses have been deposited in the Zenodo database (https://doi.org/10.5281/zenodo.7796464) [105].

## Abbreviations

PCA: Principal component analysis
WCN: Tibetan population from western China
ECN: Eastern Chinese population
AME: Americas populations
EUR: Europe populations
PC1: First principal component
PC2: Second principal component
CV: Cross validation
FSC26: *fastsimcoal26*
CADII: Carbamoyl-phosphate synthetase gene
HDM: Hengduan Mountains region
SETMAR: Histone-lysine-N-methyltransferase
DMAP1: DNA methyltransferase
*pnr*: *pannier*
2b-RAD: IIB-digest restriction site-associated DNA
*Hd*: Haplotype diversity
*π*: Nucleotide diversity
*K*: Average number of nucleotide differences
*H**o*: Heterozygosity
*F*_ST_: Pairwise differentiation
Hap1: Mitochondrial haplotypes 1
Hap2: Mitochondrial haplotypes 2
GATK: Genome Analysis Toolkit
BA3-SNPs: BayesAss3-SNPs
SFS: Site frequency spectra
