# Supplementary material for: Global patterns of genomic and phenotypic variation in the invasive harlequin ladybird
Source: BMC Biol. 2023 Jun 19;21:141. doi: 10.1186/s12915-023-01638-7 (PMC10280966; doi:10.1186/s12915-023-01638-7)
Supplement: Supplementary file 1 — Additional file 1: Table S1. Polymorphic nucleotide position of mtCOI gene defining the 22 haplotypes identified in native and non-native Harmonia axyridis populations. Table S2. Genetic diversity indices of native and non-native Harmonia axyridis populations based on 620-bp mtCOI fragment. Table S3. Pairwise FSTand migration rateof Harmonia axyridis populations from native and non-native regions. Table S4. Individual estimates the number of observed and expected homozygote sites, observed heterozygosity, and a method of moments estimate of the individual inbreeding coefficient. Table S5. Estimates of Maximum likelihood and Akaike’s information criterionacross different 3-population evolutionary demographic models. Table S6. Parameter estimates of genetic diversity, migration rate, divergence time, and population size change under the most likely 3-population topologyunder the IM model with population size changes across three major groups of Harmonia axyridis populations worldwide. Table S7. Estimates of contemporary migration between three major groups of Harmonia axyridis populations as estimated withBA3-SNPs. Table S8. Estimates of Maximum likelihood and Akaike’s information criterionacross different 4-population evolutionary demographic models. Table S9. Parameter estimates of genetic diversity, migration rate, divergence time, and population size change under the most likely 4-population topologyunder the IM model with population size changes across four major groups of Harmonia axyridis populations worldwide. Table S10. Collection information of Harmonia axyridis samples and their haplotypes from native and non-native ranges. [file 12915_2023_1638_MOESM1_ESM.docx]

**Global patterns of genomic and phenotypic variation in the invasive harlequin ladybird**

Hongran Li^1,2^, Yan Peng^2^, Yansong Wang^1^, Bryce Summerhays^3^, Xiaohan Shu^1^, Yumary Vasquez^3,4^, Hannah Vansant^3^, Christy Grenier^3^, Nicolette Gonzalez^3^, Khyati Kansagra^3^, Ryan Cartmill^3^, Edison Ryoiti Sujii ^6^, Ling Meng^1^, Xuguo Zhou^7^, Gábor L. Lövei^5^, John J Obrycki^7^, Arun Sethuraman^3,8*^ and Baoping Li^1*^

1 Department of Entomology, College of Plant Protection, Nanjing Agricultural University, Nanjing, P. R. China

2 Shenzhen Branch, Guangdong Laboratory of Lingnan Modern Agriculture, Genome Analysis Laboratory of the Ministry of Agriculture and Rural Affairs, Agricultural Genomics Institute at Shenzhen, Chinese Academy of Agricultural Sciences, Shenzhen, China

3 Department of Biological Sciences, California State University, San Marcos, CA, USA

4 Department of Life and Environmental Sciences, University of California, Merced, CA, USA

5 Department of Agroecology, Flakkebjerg Research Centre, Aarhus University; ELKH-DE Anthropocene Ecology Research Group, University of Debrecen, Hungary; Department of Zoology & Ecology, Hungarian University of Agriculture & Life Sciences, Godollo, Hungary

6 Empresa Brasileira de Pesquisa Agropecuária (Embrapa), Brasilia, DF, Brasil

7 Department of Entomology, University of Kentucky, KY, USA

8 Department of Biology, San Diego State University, CA, USA

* Co-corresponding authors, E-mails: Baoping Li: [lbp@njau.edu.cn](mailto:lbp@njau.edu.cn); Arun Sethuraman: [asethuraman@sdsu.edu](mailto:asethuraman@sdsu.edu)

**Table S1** **Polymorphic nucleotide position of *mtCOI gene* defining the 22 haplotypes identified in native and non-native *Harmonia axyridis* populations**

| Haplotype | Sequence site | | | | | | | | | | | | | | | | | | | | | | | |
| --- | --- | --- | --- | --- | --- | --- | --- | --- | --- | --- | --- | --- | --- | --- | --- | --- | --- | --- | --- | --- | --- | --- | --- | --- |
|  | 4  1 | 4  6 | 1  4  3 | 1  5  8 | 1  8  2 | 2  1  2 | 2  3  9 | 2  6  6 | 2  7  8 | 2  8  7 | 3  0  2 | 3  3  5 | 3  5  3 | 4  3  1 | 4  6  9 | 4  7  8 | 4  9  1 | 4  9  7 | 5  0  4 | 5  1  2 | 5  7  8 | 5  8  7 | 5  9  0 | 6  1  4 |
| Hap-1 | A | G | G | A | T | C | T | C | G | A | C | G | G | T | T | T | A | T | C | A | C | T | G | T |
| Hap-2 |  |  |  |  |  |  |  | T |  |  |  |  |  |  |  |  |  | A |  |  | T |  |  |  |
| Hap-3 |  |  |  |  |  |  |  |  |  | T |  |  |  |  |  |  |  |  |  |  |  |  |  |  |
| Hap-4 |  |  |  |  |  |  |  |  |  |  |  |  |  |  |  |  | T |  |  |  |  |  |  |  |
| Hap-5 |  |  |  |  |  |  |  |  |  |  |  |  |  |  |  | C |  |  |  |  |  |  |  |  |
| Hap-6 |  |  |  | G |  |  |  |  |  |  |  |  |  |  |  |  |  |  |  |  |  |  |  |  |
| Hap-7 |  |  |  |  |  |  |  |  |  |  |  |  |  |  |  |  |  |  |  |  | T |  |  |  |
| Hap-8 |  |  | A |  |  |  |  |  |  |  |  |  |  |  |  |  |  |  |  |  | T |  |  |  |
| Hap-9 |  | C |  |  |  |  |  |  |  |  |  |  |  |  |  |  |  |  |  |  |  |  |  |  |
| Hap-10 |  |  |  |  |  |  |  |  | A |  |  |  |  |  |  |  |  |  |  |  |  |  |  |  |
| Hap-11 |  |  |  |  |  |  |  |  |  |  |  | A |  |  |  |  |  |  |  |  |  |  |  |  |
| Hap-12 |  |  |  |  |  |  |  |  |  |  |  |  | A |  |  |  |  |  |  |  |  |  |  |  |
| Hap-13 |  |  |  |  |  |  | C | T |  |  |  |  |  |  |  |  |  |  |  |  |  |  |  |  |
| Hap-14 |  |  |  |  |  |  |  |  |  |  |  |  |  |  |  |  |  | A |  |  | T |  |  |  |
| Hap-15 |  |  |  |  |  |  |  |  |  |  | T |  |  |  |  |  |  |  |  |  |  |  |  |  |
| Hap-16 |  |  |  |  |  |  |  |  |  |  |  |  |  |  |  |  |  |  |  | T |  |  |  |  |
| Hap-17 |  |  | A |  |  | T |  |  | A |  |  |  |  |  |  |  |  |  |  |  | T |  |  |  |
| Hap-18 |  |  | A |  |  |  |  |  | A |  |  |  |  |  |  |  |  |  |  |  | T |  |  |  |
| Hap-19 | G |  | A |  | C | T |  |  | A |  |  |  |  |  |  |  |  |  | T |  | T |  |  |  |
| Hap-20 |  |  |  |  |  |  |  |  |  |  |  |  |  |  | G | G |  |  |  |  |  | G |  |  |
| Hap-21 |  |  |  |  |  |  |  |  |  |  |  |  |  |  |  |  |  |  |  |  |  |  | A |  |
| Hap-22 |  |  |  |  |  |  |  |  |  |  |  |  |  |  |  |  |  |  |  |  |  | G |  | A |

**Table S2** **Genetic diversity indices of native and non-native *Harmonia axyridis* populations based on 620-bp *mtCOI* fragment**

| Location/Population code* | *h* | *S* | Molecular diversity indices | | |
| --- | --- | --- | --- | --- | --- |
|  |  |  | *Hd* ± sd | π ± sd | *K* |
| Native range: mainland China | 21 | 22 | 0.458 ± 0.020 | 0.00204 ± 0.00015 | 1.26023 |
| IMG | 2 | 3 | 0.200 ± 0.154 | 0.00097 ± 0.00075 | 0.60000 |
| HBCL | 6 | 7 | 0.389 ± 0.089 | 0.00140 ± 0.00038 | 0.81935 |
| JLCC | 2 | 3 | 0.159 ± 0.094 | 0.00077 ± 0.00046 | 0.47826 |
| HLJ | 2 | 1 | 0.279 ± 0.123 | 0.00047 ± 0.00022 | 0.28947 |
| ZJHuZ | 5 | 6 | 0.488 ± 0.086 | 0.00172 ± 0.00039 | 1.06923 |
| ZJHZ | 4 | 5 | 0.518 ± 0.059 | 0.00224 ± 0.00028 | 1.38939 |
| ZJLX | 2 | 3 | 0.273 ± 0.082 | 0.00132 ± 0.00039 | 0.81935 |
| ZJLQ | 2 | 3 | 0.382 ± 0.113 | 0.00185 ± 0.00055 | 1.14706 |
| JSXZ | 8 | 9 | 0.505 ± 0.074 | 0.00197 ± 0.00034 | 1.21959 |
| JSNJ | 5 | 6 | 0.485 ± 0.069 | 0.00182 ± 0.00031 | 1.12852 |
| JSCS | 3 | 4 | 0.543 ± 0.133 | 0.00232 ± 0.00061 | 1.52381 |
| AHSZ | 5 | 6 | 0.515 ± 0.089 | 0.00211 ±0.00041 | 1.31034 |
| AHBB | 4 | 5 | 0.402 ± 0.081 | 0.00175 ± 0.00038 | 1.08549 |
| SDJN | 4 | 5 | 0.383 ± 0.073 | 0.00165 ± 0.00034 | 1.02005 |
| JXNC | 2 | 3 | 0.441 ± 0.098 | 0.00213 ± 0.00047 | 1.32353 |
| FJFZ | 4 | 3 | 0.399 ± 0.138 | 0.00083 ± 0.00033 | 0.51634 |
| SXYL | 7 | 8 | 0.686 ± 0.063 | 0.00290 ± 0.00032 | 1.79545 |
| SXDF | 5 | 5 | 0.939 ± 0.058 | 0.01261 ± 0.00444 | 7.81818 |
| SXZS | 6 | 9 | 0.933 ±0.062 | 0.00595 ± 0.00100 | 3.68889 |
| HNNY | 3 | 4 | 0.280 ±0.107 | 0.00115 ± 0.00048 | 0.71385 |
| HBYC | 3 | 4 | 0.279 ± 0.112 | 0.00154 ± 0.00091 | 0.95726 |
| HBJZ | 3 | 4 | 0.486 ± 0.124 | 0.00171 ± 0.00054 | 1.05714 |
| YNML | 3 | 3 | 0.481 ± 0.121 | 0.00160 ± 0.00049 | 0.99048 |
| YNLL | 3 | 3 | 0.407 ± 0.128 | 0.00223 ± 0.00107 | 1.38528 |
| SCLS | 4 | 5 | 0.377 ± 0.103 | 0.00127 ± 0.00041 | 0.78788 |
| GSLZ | 2 | 1 | 0.143 ± 0.119 | 0.00023 ± 0.00019 | 0.14286 |
| XZLZ | 4 | 4 | 0.558 ± 0.113 | 0.00130 ± 0.00041 | 0.80526 |
| GZGY | 6 | 6 | 0.641 ± 0.130 | 0.00254 ± 0.00080 | 1.57516 |
| XJWL | 6 | 8 | 0.621 ± 0.109 | 0.00244 ± 0.00057 | 1.51053 |
|  |  |  |  |  |  |
| Non-native range: North America | 5 | 42 | 0.370 ± 0.055 | 0.00183 ± 0.00069 | 1.13179 |
| USATN | 1 | 0 | 0 | 0 | 0 |
| USAIA | 3 | 2 | 0.562 ± 0.143 | 0.00735 ± 0.00458 | 4.55238 |
| USANY | 1 | 0 | 0 | 0 |  |
| USAIL | 2 | 1 | 0.337 ± 0.110 | 0.00054 ± 0.00018 | 0.33684 |
| USAMN | 2 | 1 | 0.352 ± 0.131 | 0.00185 ± 0.00096 | 1.14286 |
| USAKS | 1 | 0 | 0 | 0 |  |
| USAKY | 3 | 3 | 0.339 ± 0.115 | 0.00081 ± 0.00032 | 0.50427 |
|  |  |  |  |  |  |
| Non-native range: South America | 5 | 6 | 0.689 ± 0.096 | 0.00235 ± 0.00051 | 1.45299 |
| BRGO | 5 | 6 | 0.689 ± 0.096 | 0.00235 ± 0.00051 | 1.45299 |
| Non-native range: Europe | 2 | 7 | 0.147 ± 0.066 | 0.00048 ± 0.00030 | 0.30044 |
| PLKK | 2 | 1 | 0.100 ± 0.088 | 0.00016 ± 0.00014 | 0.10000 |
| GMDD | 2 | 1 | 0.182 ± 0.144 | 0.00029 ± 0.00023 | 0.18182 |
| GMPD | 1 | 0 | 0 | 0 | 0 |
| ATVN | 1 | 0 | 0.524 ± 0.209 | 0.00261 ± 0.00145 | 1.61905 |
| All populations | 22 | 24 | 0.438 ± 0019 | 0.00194 ± 0.00015 | 1.19433 |

*h*, number of haplotypes; *S* number of polymorphic (segregating) sites; *Hd*, haplotype diversity; *π*, nucleotide diversity; *K*, average number of nucleotide differences.

**Table S3** **Pairwise *F*_ST_ (below diagonal) and migration rate (*N*_m_) (above diagonal) of *Harmonia axyridis* populations from native and non-native regions**

| **Place of population** | **Native region: mainland China** | **Non-native region: North America** | **Non-native region: South America** | **Non-native region: Europe** |
| --- | --- | --- | --- | --- |
| Native range: mainland China |  | 2.75734 | 3.30973 | 2.03603 |
| Non-native range: North America | 0.08313 |  | 231.23148 | 16.42779 |
| Non-native range: South America | 0.07023 | 0.00108 |  | 24.75 |
| Non-native range: Europe | 0.10936 | 0.01499 | 0.01000 |  |

**Table S4 Individual estimates the number of observed and expected homozygote sites, observed heterozygosity, and a method of moments estimate of the individual inbreeding coefficient (F).**

| **Individual** | **Homozygote Sites** | **Expected Homozygous Sites** | **Heterozygosity** | **Total # of Sites** | **Inbreeding Coefficient (F)** |
| --- | --- | --- | --- | --- | --- |
| ATVN1 | 6101 | 5625.9 | 0.174648268 | 7392 | 0.269 |
| ATVN2 | 6143 | 5663.2 | 0.174660755 | 7443 | 0.26957 |
| ATVN3 | 6183 | 5643.8 | 0.166486924 | 7418 | 0.3039 |
| ATVN4 | 5952 | 5582.3 | 0.188326742 | 7333 | 0.21116 |
| ATVN5 | 6132 | 5641.9 | 0.172581298 | 7411 | 0.27701 |
| ATVN6 | 6118 | 5484.5 | 0.150513746 | 7202 | 0.36884 |
| Ap | 122 | 90.2 | 0 | 122 | 1 |
| BREM1 | 6068 | 5597.8 | 0.175543478 | 7360 | 0.26684 |
| BREM2 | 6108 | 5572.8 | 0.165801694 | 7322 | 0.30598 |
| BREM3 | 6155 | 5520.4 | 0.152554041 | 7263 | 0.36417 |
| BREM4 | 6066 | 5536.7 | 0.167101469 | 7283 | 0.30309 |
| BREM5 | 6133 | 5565.6 | 0.161356488 | 7313 | 0.32472 |
| FJFZ1 | 5701 | 5270 | 0.174007534 | 6902 | 0.26408 |
| FJFZ2 | 6265 | 5668.1 | 0.159060403 | 7450 | 0.33497 |
| FJFZ3 | 6283 | 5718.8 | 0.163159297 | 7508 | 0.31532 |
| FJFZ4 | 6184 | 5585.8 | 0.156918882 | 7335 | 0.342 |
| FJFZ5 | 6143 | 5441.9 | 0.140478522 | 7147 | 0.41117 |
| FJFZ6 | 6217 | 5485.3 | 0.137246739 | 7206 | 0.42523 |
| GMDD1 | 6095 | 5575.7 | 0.168258734 | 7328 | 0.29633 |
| GMDD2 | 5999 | 5492.6 | 0.16807655 | 7211 | 0.29467 |
| GMDD3 | 6121 | 5610.4 | 0.170708576 | 7381 | 0.28838 |
| GMDD4 | 5196 | 4389.6 | 0.092877095 | 5728 | 0.6025 |
| GMDD5 | 6141 | 5620.1 | 0.167660613 | 7378 | 0.29633 |
| GSLZ1 | 6157 | 5589.7 | 0.16162854 | 7344 | 0.32338 |
| GSLZ2 | 6199 | 5638.8 | 0.163653535 | 7412 | 0.31594 |
| GSLZ3 | 6247 | 5681.7 | 0.163273507 | 7466 | 0.31681 |
| GSLZ4 | 6195 | 5656.4 | 0.165993538 | 7428 | 0.30403 |
| GSLZ5 | 6221 | 5584.7 | 0.152336831 | 7339 | 0.36273 |
| GSLZ6 | 6177 | 5626.8 | 0.164027609 | 7389 | 0.31223 |
| HBYC1 | 6169 | 5634.9 | 0.166801729 | 7404 | 0.3019 |
| HBYC2 | 6187 | 5658.8 | 0.167854741 | 7435 | 0.29739 |
| HBYC3 | 6219 | 5637.7 | 0.160275452 | 7406 | 0.32874 |
| HBYC4 | 6251 | 5661.7 | 0.159924741 | 7441 | 0.33121 |
| HBYC5 | 6279 | 5596.3 | 0.146990898 | 7361 | 0.38687 |
| HBYC6 | 6168 | 5614.5 | 0.164340875 | 7381 | 0.31335 |
| HNNY1 | 6251 | 5626.7 | 0.154356061 | 7392 | 0.35364 |
| HNNY2 | 6155 | 5656.1 | 0.171601615 | 7430 | 0.28124 |
| HNNY3 | 6258 | 5657 | 0.158644797 | 7438 | 0.33747 |
| HNNY4 | 6233 | 5625.5 | 0.157133198 | 7395 | 0.3433 |
| HNNY5 | 6191 | 5579.8 | 0.155388813 | 7330 | 0.34922 |
| HNNY6 | 6191 | 5640.2 | 0.165183387 | 7416 | 0.31017 |
| JLCC1 | 6188 | 5603.2 | 0.15923913 | 7360 | 0.33287 |
| JLCC2 | 6226 | 5583.1 | 0.150961407 | 7333 | 0.36739 |
| JLCC3 | 6199 | 5600.4 | 0.157630113 | 7359 | 0.34037 |
| JLCC4 | 6146 | 5600.4 | 0.164832178 | 7359 | 0.31025 |
| JLCC5 | 6224 | 5617.2 | 0.156639566 | 7380 | 0.34423 |
| JLCC6 | 6274 | 5630.2 | 0.151703624 | 7396 | 0.36458 |
| JXNC1 | 6254 | 5591.1 | 0.148652328 | 7346 | 0.37776 |
| JXNC2 | 6179 | 5625.5 | 0.163756936 | 7389 | 0.31388 |
| JXNC3 | 6175 | 5589.4 | 0.159863946 | 7350 | 0.3326 |
| JXNC4 | 6159 | 5611.3 | 0.163747454 | 7365 | 0.31232 |
| JXNC5 | 6267 | 5591.2 | 0.146882657 | 7346 | 0.38512 |
| JXNC6 | 6205 | 5607.3 | 0.158187492 | 7371 | 0.3389 |
| LNSY1 | 6201 | 5632.8 | 0.162366608 | 7403 | 0.32099 |
| LNSY2 | 6136 | 5610.2 | 0.167548501 | 7371 | 0.29861 |
| LNSY3 | 6254 | 5596.1 | 0.149231397 | 7351 | 0.37489 |
| LNSY4 | 6267 | 5639.4 | 0.154251012 | 7410 | 0.35447 |
| LNSY5 | 6243 | 5645.1 | 0.158625337 | 7420 | 0.33685 |
| LNSY6 | 6228 | 5656 | 0.162002153 | 7432 | 0.32207 |
| PLKK1 | 6086 | 5606.3 | 0.173434741 | 7363 | 0.27305 |
| PLKK2 | 6173 | 5644.2 | 0.167610572 | 7416 | 0.29845 |
| PLKK3 | 6184 | 5688.4 | 0.17293032 | 7477 | 0.27709 |
| PLKK4 | 6104 | 5575 | 0.16668942 | 7325 | 0.30229 |
| PLKK5 | 6150 | 5635.5 | 0.169704334 | 7407 | 0.29045 |
| PLKK6 | 6147 | 5597.9 | 0.164809783 | 7360 | 0.31163 |
| SCLS1 | 6173 | 5534.2 | 0.150543553 | 7267 | 0.36866 |
| SCLS2 | 6151 | 5588.4 | 0.161874915 | 7339 | 0.32138 |
| SCLS3 | 6275 | 5645.3 | 0.153856526 | 7416 | 0.35561 |
| SCLS4 | 6123 | 5587.4 | 0.165576451 | 7338 | 0.30596 |
| SCLS5 | 6200 | 5608.1 | 0.158294868 | 7366 | 0.3367 |
| SCLS6 | 6229 | 5585.7 | 0.151709111 | 7343 | 0.36606 |
| SDLS1 | 6293 | 5638 | 0.150283554 | 7406 | 0.37046 |
| SDLS2 | 6185 | 5658.5 | 0.167675952 | 7431 | 0.29706 |
| SDLS3 | 6200 | 5639.6 | 0.163744268 | 7414 | 0.31583 |
| SDLS4 | 6202 | 5652.8 | 0.165949435 | 7436 | 0.30799 |
| SDLS5 | 6178 | 5624.7 | 0.164231602 | 7392 | 0.31308 |
| SDLS6 | 6272 | 5596.9 | 0.147014824 | 7353 | 0.38445 |
| SXYL1 | 6240 | 5664.9 | 0.161403037 | 7441 | 0.3238 |
| SXYL2 | 6230 | 5622.4 | 0.15719697 | 7392 | 0.34337 |
| SXYL3 | 6262 | 5653.5 | 0.157200538 | 7430 | 0.34252 |
| SXYL4 | 6226 | 5616.1 | 0.156482861 | 7381 | 0.34558 |
| SXYL5 | 6211 | 5623.8 | 0.160221741 | 7396 | 0.33134 |
| SXYL6 | 6155 | 5635.7 | 0.168467982 | 7402 | 0.294 |
| USAAR1 | 6142 | 5643.1 | 0.171567305 | 7414 | 0.28172 |
| USAAR2 | 6109 | 5652.2 | 0.177792732 | 7430 | 0.25695 |
| USAAR3 | 6106 | 5662.4 | 0.179742074 | 7444 | 0.24899 |
| USAAR4 | 6128 | 5661.6 | 0.176233365 | 7439 | 0.2624 |
| USAAR5 | 6077 | 5606.6 | 0.174993212 | 7366 | 0.26738 |
| USAAR6 | 6137 | 5620.3 | 0.169778139 | 7392 | 0.29165 |
| USAIA1 | 6097 | 5614.3 | 0.173624288 | 7378 | 0.27368 |
| USAIA2 | 6188 | 5719.8 | 0.175922227 | 7509 | 0.26166 |
| USAIA3 | 6074 | 5729.4 | 0.192609331 | 7523 | 0.19213 |
| USAIA4 | 6037 | 5669.8 | 0.18966443 | 7450 | 0.20628 |
| USAIA5 | 6008 | 5545.5 | 0.175518046 | 7287 | 0.26558 |
| USAIA6 | 6097 | 5512.8 | 0.158454106 | 7245 | 0.33727 |
| USAIL1 | 6019 | 5632.4 | 0.186511691 | 7399 | 0.21885 |
| USAIL2 | 6140 | 5599.6 | 0.165987503 | 7362 | 0.30662 |
| USAIL3 | 6071 | 5629.7 | 0.179705445 | 7401 | 0.24914 |
| USAIL4 | 6092 | 5627.1 | 0.17631152 | 7396 | 0.26282 |
| USAIL5 | 6042 | 5615 | 0.180634662 | 7374 | 0.24276 |
| USAIL6 | 6187 | 5660.2 | 0.168302191 | 7439 | 0.29615 |
| USAIN1 | 6112 | 5590.6 | 0.168548497 | 7351 | 0.29619 |
| USAIN2 | 6061 | 5635.6 | 0.181056614 | 7401 | 0.24095 |
| USAIN3 | 6093 | 5613.4 | 0.173942516 | 7376 | 0.2721 |
| USAIN4 | 6053 | 5602.5 | 0.178028246 | 7364 | 0.25573 |
| USAIN5 | 6094 | 5646.5 | 0.179148707 | 7424 | 0.25175 |
| USAIN6 | 6076 | 5643.2 | 0.180358829 | 7413 | 0.24454 |
| USAKS1 | 6109 | 5621.6 | 0.172558581 | 7383 | 0.27673 |
| USAKS2 | 6110 | 5667.5 | 0.179865772 | 7450 | 0.24824 |
| USAKS3 | 6100 | 5638.9 | 0.177232263 | 7414 | 0.25975 |
| USAKS4 | 6103 | 5591.5 | 0.169320811 | 7347 | 0.29136 |
| USAKS5 | 6109 | 5641.7 | 0.17590719 | 7413 | 0.26382 |
| USAKS6 | 6075 | 5619.4 | 0.17727519 | 7384 | 0.2582 |
| USAKY1 | 6085 | 5638.5 | 0.179255463 | 7414 | 0.25146 |
| USAKY2 | 6005 | 5640 | 0.189389849 | 7408 | 0.20646 |
| USAKY3 | 6073 | 5603.4 | 0.175648161 | 7367 | 0.26629 |
| USAKY4 | 6010 | 5645.8 | 0.189043314 | 7411 | 0.20632 |
| USAKY5 | 6080 | 5618.2 | 0.176821013 | 7386 | 0.26123 |
| USAKY6 | 6052 | 5624.4 | 0.181387799 | 7393 | 0.24177 |
| USAMN1 | 6041 | 5538.1 | 0.170306277 | 7281 | 0.28856 |
| USAMN2 | 6087 | 5645.2 | 0.179539021 | 7419 | 0.24905 |
| USAMN3 | 6085 | 5544.8 | 0.164263151 | 7281 | 0.31113 |
| USAMN4 | 6088 | 5597.4 | 0.17215121 | 7354 | 0.27928 |
| USAMN5 | 6095 | 5582.5 | 0.169505382 | 7339 | 0.29178 |
| USAMN6 | 6093 | 5552.4 | 0.165228113 | 7299 | 0.30952 |
| USANY1 | 6062 | 5560.3 | 0.170384563 | 7307 | 0.28723 |
| USANY2 | 6112 | 5596.6 | 0.16900068 | 7355 | 0.29309 |
| USANY3 | 5653 | 5070.1 | 0.150435828 | 6654 | 0.36802 |
| USANY4 | 6140 | 5592.2 | 0.16496668 | 7353 | 0.31112 |
| USANY5 | 5988 | 5594.8 | 0.185306122 | 7350 | 0.22402 |
| USATN1 | 6105 | 5672 | 0.180866765 | 7453 | 0.24312 |
| USATN2 | 6082 | 5668.7 | 0.183624161 | 7450 | 0.23203 |
| USATN3 | 5954 | 5554 | 0.183600713 | 7293 | 0.23004 |
| USATN4 | 6039 | 5661.2 | 0.188197338 | 7439 | 0.21253 |
| USATN5 | 6045 | 5599.8 | 0.17822186 | 7356 | 0.25349 |
| USATN6 | 6080 | 5650.9 | 0.181034483 | 7424 | 0.24202 |
| USAVA1 | 6085 | 5565.7 | 0.167692518 | 7311 | 0.29753 |
| USAVA2 | 6168 | 5673.2 | 0.172191652 | 7451 | 0.27833 |
| USAVA3 | 6112 | 5654.9 | 0.177056685 | 7427 | 0.25793 |
| USAVA4 | 6107 | 5663 | 0.179166667 | 7440 | 0.24984 |
| USAVA5 | 6071 | 5655.4 | 0.183127018 | 7432 | 0.23392 |
| USAVA6 | 6096 | 5717 | 0.188498403 | 7512 | 0.21112 |
| XJWL1 | 6139 | 5604.7 | 0.16634981 | 7364 | 0.30368 |
| XJWL2 | 6181 | 5638.4 | 0.165180983 | 7404 | 0.30731 |
| XJWL3 | 6204 | 5629.7 | 0.161281601 | 7397 | 0.32497 |
| XJWL4 | 6144 | 5619.4 | 0.16826858 | 7387 | 0.29677 |
| XJWL5 | 6186 | 5633.3 | 0.164505673 | 7404 | 0.31215 |
| XJWL6 | 6241 | 5656.7 | 0.159913851 | 7429 | 0.3297 |
| XZLZ1 | 6156 | 5548 | 0.155671376 | 7291 | 0.34881 |
| XZLZ2 | 6222 | 5468.6 | 0.133788111 | 7183 | 0.43945 |
| XZLZ3 | 6134 | 5500.6 | 0.151120952 | 7226 | 0.36711 |
| XZLZ4 | 6191 | 5520 | 0.146657478 | 7255 | 0.38676 |
| XZLZ5 | 6163 | 5453.6 | 0.139846476 | 7165 | 0.4145 |
| XZLZ6 | 6225 | 5458 | 0.132887589 | 7179 | 0.44568 |
| YNQJ1 | 6202 | 5612.1 | 0.159164859 | 7376 | 0.33441 |
| YNQJ2 | 6217 | 5633.9 | 0.159524131 | 7397 | 0.33071 |
| YNQJ3 | 6156 | 5586 | 0.160964972 | 7337 | 0.32555 |
| YNQJ4 | 6181 | 5624.9 | 0.163146493 | 7386 | 0.31575 |
| YNQJ5 | 6207 | 5604.1 | 0.156886716 | 7362 | 0.34296 |
| YNQJ6 | 6174 | 5549.2 | 0.153318705 | 7292 | 0.3585 |

**Table S5.** **Estimates of Maximum likelihood and Akaike’s information criterion (AIC) across different 3-population evolutionary demographic models tested to fit the empirical distribution of site frequency spectra across between three major groups (America, Europe, and Asia) of *Harmonia axyridis*** **populations.**

| **Model** | **Topology** | **Maximum Observed Likelihood** | **Delta L** | **AIC** |
| --- | --- | --- | --- | --- |
| No Migration | t1 | − 24252.973 | 12413.421 | 111705.0682 |
| No Migration | t2 | − 24438.134 | 12598.582 | 112557.7661 |
| No Migration | t3 | − 39998.839 | 28159.287 | 184217.4608 |
| No Migration | t4 | − 24357.385 | 12517.833 | 112181.9032 |
| No Migration + growth | t1 | -24275.705 | 12436.153 | 111823.7529 |
| No Migration + growth | t2 | -24297.934 | 12458.382 | 111926.1212 |
| No Migration + growth | t3 | -24329.579 | 12490.027 | 112069.8518 |
| No Migration + growth | t4 | -24313.337 | 12473.785 | 111991.0547 |
| IM | t1 | − 21432.088 | 9592.536 | 98730.41268 |
| IM | t2 | − 22396.246 | 10556.694 | 103170.5244 |
| IM | t3 | − 22309.958 | 10470.406 | 102773.1534 |
| IM | t4 | − 22296.253 | 10456.701 | 102704.0396 |
| IM + growth | t1 | − 20561.810 | 8722.258 | 94736.63438 |
| IM + growth | t2 | − 24326.081 | 28349.47 | 185123.2859 |
| IM + growth | t3 | − 24285.138 | 28444.945 | 185562.9645 |
| IM + growth | t4 | − 24323.537 | 28443.626 | 185546.8903 |

Note: Tested models include: (1) isolation without migration, (2) isolation without migration, with changing population sizes, (3) isolation with asymmetric migration, and (4) isolation with asymmetric migration, with changing population sizes each under four topologies: (1) t1: ((Asia, Europe), MRCA1),USA) MRCA2, (2) t2: ((Asia,USA), MRCA1), Europe) MRCA2, (3) t3: ((Europe, USA), MRCA1), Asia)MRCA2, and (4) t4: (Asia, Europe, USA) MRCA. The best supported model (IM with populations size change, t1) is marked in red.

**Table S6.** **Parameter estimates of genetic diversity, migration rate, divergence time, and population size change under the most likely 3-population topology (t1) under the IM model with population size changes across three major groups of *Harmonia axyridis*** **populations worldwide.**

| **Parameter** | **Estimate** | **CI lower** | **CI upper** | **Std. Error** |
| --- | --- | --- | --- | --- |
| NAncAll | 1971571.34 | 1970330 | 1972812 | 625.4615 |
| Nanc01 | 1.64E+06 | 1592567 | 1689825 | 24507.96 |
| Npop0 (Asia) | 1.46E+06 | 1405134 | 1512869 | 27147.96 |
| Npop1 (Europe) | 1036211.10 | 955242.1 | 1117180 | 40806.53 |
| Npop2 (Americas) | 720962.14 | 609163.29 | 832761 | 56344.06 |
| TDIV01 | 1.44E+01 | 13.11693 | 15.6430723 | 0.63656 |
| TDIV2_ANC1 | 45.290000 | 42.549818 | 48.03018 | 1.380989 |
| MIG01 | 6.73E-08 | 4.13E-08 | 9.32E-08 | 1.31E-08 |
| MIG10 | 1.05E-07 | 4.04E-08 | 1.69E-07 | 3.23E-08 |
| MIG20 | 5.83054E-06 | − 2.420651E-06 | 1.408173e-05 | 4.16E+00 |
| MIG02 | 1.20E-07 | 4.70E-08 | 1.94E-07 | 3.698503E-08 |
| MIG12 | 8.30E-08 | 4.01E-08 | 1.26E-07 | 2.162887E-08 |
| MIG21 | 5.01863E-06 | − 2.544615E-06 | 1.258188e-05 | 3.81E-06 |
| MIG2A | 6.04E-06 | − 4.196223E-07 | 1.249402e-05 | 3.25E-06 |
| MIGA2 | 1.22E-07 | 6.54E-08 | 1.79E-07 | 2.86E-08 |
| GrowthP0 | − 0.073044464 | − 0.088499 | − 0.057589845 | 0.007789 |
| GrowthP1 | − 6.81E-02 | − 0.079876 | − 0.056371994 | 0.005923 |
| GrowthP2 | − 2.36E-01 | − 0.30827 | − 0.16412779 | 0.036322 |

Note: 95% confidence intervals were constructed across 100 bootstrap replicates in the FSC26.

**Table S7. Estimates of contemporary migration between three major groups of *Harmonia axyridis*** **populations as estimated with BA3-SNPs**

| Summary statistic | m[0][0] | m[0][1] | m[0][2] | m[1][0] | m[1][1] | m[1][2] | m[2][0] | m[2][1] | m[2][2] |
| --- | --- | --- | --- | --- | --- | --- | --- | --- | --- |
| mean | 0.9667 | 0.0165 | 0.0169 | 4.89E-03 | 0.9902 | 4.89E-03 | 4.05E-03 | 4.16E-03 | 0.9918 |
| 95% high probabily density (HPD) interval | [0.9252, 0.9987] | [7.7599E-7, 0.0476] | [3.1783E-7, 0.0498] | [6.3436E-7, 0.0148] | [0.9768, 0.9997] | [7.5411E-7, 0.0149] | [1.9297E-6, 0.0121] | [1.7161E-7, 0.0124] | [0.9808, 0.9999] |

Note: 1, Americas; 2, Asia; 0, Europe.

**Table S8.** **Estimates of Maximum likelihood and Akaike’s information criterion (AIC) across different 4-population evolutionary demographic models tested to fit the empirical distribution of site frequency spectra across between four major groups (America, Europe, Eastern China, and Western China) of *Harmonia axyridis*** **populations.**

| **Model** | **Topology** | **Maximum Observed Likelihood** | **Delta L** | **AIC** |
| --- | --- | --- | --- | --- |
| No Migration | t1 | -1041.492 | 1041.384 | 4818.247 |
| No Migration + growth | t1 | -222.299 | 222.191 | 1049.724 |
| IM | t1 | -1041.718 | 1041.61 | 4853.288 |
| IM + growth | t1 | -220.642 | 220.534 | 1082.094 |

Note: The only 4-population topology tested used the most likely 3-population model estimated with fastsimcoal26, and involved the earliest divergence of Eastern and Western Chinese populations, followed by the split of the American populations from the Eastern Chinese population, and a hybrid establishment of the European population from America and Eastern China.

**Table S9. Parameter estimates of genetic diversity, migration rate, divergence time, and population size change under the most likely 4-population topology (t1) under the IM model with population size changes across four major groups of *Harmonia axyridis* populations worldwide.**

| **Parameter** | **Mean** | **Stdev** | **Confidence** | **Upper CI** | **Lower CI** |
| --- | --- | --- | --- | --- | --- |
| N_POP0 | 1.97E+06 | 5640.66927 | 1105.55086 | 1971073.2 | 1968862.1 |
| N_POP1 | 1.13E+06 | 398617.541 | 78127.6023 | 1204866.6 | 1048611.4 |
| N_POP2 | 1.03E+06 | 429240.02 | 84129.4979 | 1111575.01 | 943316.012 |
| N_POP3 | 1.05E+06 | 393708.895 | 77165.5254 | 1124859.78 | 970528.725 |
| TMIX | 12.86 | 4.06517608 | 0.79675987 | 13.6567599 | 12.0632401 |
| TDIV123 | 23.92 | 2.53731744 | 0.49730508 | 24.4173051 | 23.4226949 |
| TDIV0_ANC123 | 42.85 | 6.5 | 1.27397659 | 44.1239766 | 41.5760234 |
| 1m01 | 0.02246785 | 0.05466053 | 0.01071327 | 0.03318112 | 0.01175458 |
| 1m02 | 0.01352083 | 0.03858845 | 0.0075632 | 0.02108402 | 0.00595763 |
| 1m03 | 0.02336726 | 0.05657282 | 0.01108807 | 0.03445533 | 0.01227919 |
| 1m10 | 0.03178973 | 0.06998641 | 0.01371708 | 0.04550682 | 0.01807265 |
| 1m12 | 0.02149533 | 0.05007033 | 0.00981361 | 0.03130894 | 0.01168173 |
| 1m13 | 0.02043427 | 0.05809211 | 0.01138584 | 0.03182012 | 0.00904843 |
| 1m20 | 0.0357222 | 0.0687137 | 0.01346764 | 0.04918983 | 0.02225456 |
| 1m21 | 0.02742495 | 0.08348391 | 0.01636255 | 0.0437875 | 0.01106241 |
| 1m23 | 0.02421237 | 0.04673066 | 0.00915904 | 0.03337141 | 0.01505333 |
| 1m30 | 0.01931246 | 0.04008578 | 0.00785667 | 0.02716913 | 0.01145579 |
| 1m31 | 0.01113227 | 0.02187214 | 0.00428686 | 0.01541913 | 0.00684541 |
| 1m32 | 0.01305354 | 0.02733853 | 0.00535825 | 0.0184118 | 0.00769529 |
| 2m01 | 0.03062667 | 0.05532769 | 0.01084403 | 0.0414707 | 0.01978264 |
| 2m03 | 0.0204131 | 0.03664725 | 0.00718273 | 0.02759583 | 0.01323037 |
| 2m10 | 0.03159413 | 0.07835727 | 0.01535774 | 0.04695187 | 0.01623639 |
| 2m13 | 0.01884268 | 0.04364677 | 0.00855461 | 0.02739729 | 0.01028807 |
| 2m30 | 0.02196292 | 0.05145729 | 0.01008544 | 0.03204836 | 0.01187747 |
| 2m31 | 0.03663458 | 0.08712433 | 0.01707605 | 0.05371064 | 0.01955853 |
| 3m01 | 0.03001587 | 0.0635263 | 0.01245093 | 0.0424668 | 0.01756494 |
| 3m10 | 0.04395555 | 0.0819239 | 0.01605679 | 0.06001234 | 0.02789877 |
| N0atSPLIT | 20587423.3 | 97674.2118 | 19143.7937 | 20606567.1 | 20568279.5 |
| N1atSPLIT | 511943.95 | 307970.946 | 60361.1962 | 572305.146 | 451582.754 |
| N2atSPLIT | 520879.39 | 325425.011 | 63782.1301 | 584661.52 | 457097.26 |
| N3atSPLIT | 459503.58 | 276485.678 | 54190.197 | 513693.777 | 405313.383 |
| GrowthP0 | 0.05615092 | 0.00940222 | 0.0018428 | 0.05799372 | 0.05430812 |
| GrowthP1 | -0.0378437 | 0.0205944 | 0.00403643 | -0.0338072 | -0.0418801 |
| GrowthP2 | -0.0698385 | 0.0537829 | 0.01054126 | -0.0592972 | -0.0803797 |
| GrowthP3 | -0.0392151 | 0.01988523 | 0.00389743 | -0.0353177 | -0.0431125 |

**Table S10 Collection information of *Harmonia axyridis* samples and their haplotypes from native and non-native ranges**

| **Region** | **Code** | **Location (city, province or state, country)** | **Longitude (°E/W)** | **Latitude (°N/S)** | **Date** | **Habitat** | **2b-**  **RAD** | **mtCOI** | **Hap1** | **Hap2** | **Others** |
| --- | --- | --- | --- | --- | --- | --- | --- | --- | --- | --- | --- |
| Native range: mainland China | NMG | Wulanchabu, Inner mongolia | 113.85 | 40.86 | 2017.09 | Potato field | / | 10 | 8 | 1 | 1 |
|  | HBCL | Changli, Hebei | 119.17 | 39.72 | 2017.05 | - | / | 45 | 35 | 5 | 4 |
|  | JLCC^🕇^ | Changchun, Jilin | 125.40 | 43.81 | 2017 | Mountain | 6 | 24 | 22 | 2 | 0 |
|  | LNSY | Shenyang, Liaoning | 123.57 | 41.83 | 2018.10 | - | 6 | / | / | / | / |
|  | HLJ^🕇^ | Harbin, Heilongjiang | 126.92 | 45.78 | 2017 | Mountain | / | 20 | 17 | 0 | 2 |
|  | ZJHuZ | Huzhou, Zhejiang | 126.02 | 30.86 | 2017.05 | Reed | / | 40 | 28 | 6 | 6 |
|  | ZJHZ | Hangzhou, Zhejiang | 120.05 | 30.25 | 2017.05 | Shrub | / | 50 | 32 | 14 | 4 |
|  | ZJLX | Lanxi, Zhejiang | 119.56 | 29.31 | 2017.05 | Vegetable field | / | 38 | 32 | 6 | 0 |
|  | ZJLQ | Longquan, Zhejiang | 119.14 | 28.08 | 2017.05 | Peach | / | 17 | 13 | 4 | 0 |
|  | JSXZ | Xuzhou, Jiangsu | 117.29 | 34.28 | 2017.05 | - | / | 50 | 34 | 10 | 6 |
|  | JSNJ | Nanjing, Jiangsu | 118.63 | 32.03 | 2017.05 | fava bean farmland | / | 63 | 44 | 11 | 6 |
|  | JSCS | Changshu, Jiangsu | 120.92 | 31.59 | 2017.05 | - | / | 15 | 10 | 3 | 1 |
|  | AHSZ | Suzhou, Anhui | 117.25 | 33.68 | 2017.05 | Reed | / | 30 | 20 | 7 | 3 |
|  | AHBB | Bengbu, Anhui | 117.45 | 32.91 | 2017.05 | weed (feverfew) | / | 51 | 39 | 7 | 2 |
|  | SDJN | Jinan, Shandong | 116.81 | 36.60 | 2017.05 | Maize field | / | 57 | 44 | 9 | 3 |
|  | SDLS | Liangshan, Shandong | 115.92 | 35.90 | 2018.10 | Maize field | 6 | - | - | - | - |
|  | JXNC | Nanchang, Jiangxi | 116.01 | 28.68 | 2018.06 | Citrus orchard | 6 | 17 | 12 | 5 | 0 |
|  | FJFZ | Fuzhou, Fujian | 119.30 | 26.07 | 2018.07 |  | 6 | 18 | 14 | 2 | 2 |
|  | SXYL | Yangling, Shanxi | 108.08 | 34.26 | 2017.05 | Shrub | 6 | 33 | 16 | 10 | 6 |
|  | SXDF | Danfeng, Shanxi | 110.30 | 33.88 | 2014.06 | Wheat field | / | 12 | 0 | 0 | 8 |
|  | SXZS | Zhashui, Shanxi | 109.38 | 34.57 | 2014.06 | Wheat field | / | 10 | 2 | 2 | 5 |
|  | HNNY | Nanyang, Henan | 111.03 | 33.24 | 2017.06 | Pennisetum | 6 | 26 | 22 | 3 | 1 |
|  | HBYC | Yichang, Hubei | 111.76 | 30.43 | 2018.07 | Pear and Peach tree | 6 | 27 | 23 | 1 | 1 |
|  | HBJZ | Jingzhou, Hubei | 112.15 | 30.35 | 2018.06 | Weed | / | 21 | 16 | 3 | 0 |
|  | YNML | Malong, Yunnan | 103.67 | 25.03 | 2018.07 | Weed | 6 | 21 | 15 | 3 | 1 |
|  | YNLL^🕇^ | Luliang, Yunnan | 103.58 | 25.43 | 2018 | Mountain | / | 22 | 17 | 2 | 2 |
|  | SCLS | Liangshan, Sichuan | 102.24 | 26.65 | 2018.08 | Weed | 6 | 33 | 26 | 3 | 3 |
|  | GSLZ | Lanzhou, Gansu | 103.69 | 36.09 | 2018.10 | Crop land | 6 | 14 | 13 | 0 | 1 |
|  | XZLZ | Linzhi, Xizang | 94.36 | 29.63 | 2019.08 | Weed | 6 | 20 | 13 | 1 | 6 |
|  | GZGY | Guiyang, Guizhou | 106.63 | 26.39 | 2018.06 | Maize field | / | 18 | 11 | 1 | 4 |
|  | XJWL | Wulumuqi, Xinjiang | 43.98 | 87.5 | 2019.08 |  | 6 | 20 | 8 | 3 | 9 |
|  |  |  |  |  |  |  |  |  |  |  |  |
| Non-native range: North America | USATN | Knoxville, Tennessee | 86.82 | 36.02 | 2018.08 | Farmland | 6 | 27 | 27 | 0 | 0 |
|  | USAIA | Ames, Iowa | 93.37 | 42.01 | 1996 | - | / | 15 | 9 | 0 | 4 |
|  | USANY | Fayetteville, New York | 76.01 | 43.03 | 2017.07 | Farmland | 5 | 5 | 5 | 0 | 0 |
|  | USAIL | Leroy, Illinois | 88.72 | 40.31 | 2017.09 | Farmland | 6 | 20 | 16 | 0 | 4 |
|  | USAMN | Minneapolis, Minnesota | 93.29 | 44.93 | 2017.08 | Farmland | 6 | 21 | 17 | 0 | 1 |
|  | USAKS | Lawrence, Kansas | 95.21 | 38.90 | 2017.05 | Weed | 6 | 8 | 8 | 0 | 0 |
|  | USAKY | Lexington, Kentucky | 84.51 | 38.01 | 2017.09 | Sorghum | 6 | 27 | 22 | 0 | 3 |
|  | USAAR | Foreman, Arkansas | 94.39 | 33.72 | 1996 | NA | 6 |  |  |  |  |
|  | USAVA | - | - | - | 1994 | NA | 6 |  |  |  |  |
|  | USAIA | Ames, Iowa | 93.37 | 42.01 | 1996 | NA | 6 |  |  |  |  |
|  | USAIN | Clinton, Indiana | 87.40 | 39.65 | 2017 | NA | 6 |  |  |  |  |
| Non-native range: South America | BREM | Brasilia, Goiás | 47.90 | 15.73 | 2018.05 | NA | 5 | 27 | 15 | 0 | 8 |
|  |  |  |  |  |  |  |  |  |  |  |  |
| Non-native range: Europe | PLKK | Krakow, Poland | 19.93 | 50.06 | 2018 | NA | 6 | 20 | 19 | 0 | 1 |
|  | GMPD | Potsdam, Germany | 13.07 | 52.39 | 2018 | NA | / | 15 | 15 | 0 | 0 |
|  | GMDD | Dresden, Germany | 13.73 | 51.05 | 2018 | NA | 5 | 11 | 10 | 0 | 1 |
|  | ATVN | Vienna, Austria | 16.38 | 48.22 | 2018 | NA | 6 | 7 | 5 |  | 0 |
|  |  |  |  |  |  |  |  |  |  |  |  |
| Total | 41 populations | |  |  |  |  | 159 | 1025 |  |  |  |

Note: The haplotype was identified by having at least two identical sequences. Others: Haplotype 3-22. **^🕇^**indicating the overwintering populations
