# Supplementary material for: Global patterns of genomic and phenotypic variation in the invasive harlequin ladybird
Source: BMC Biol. 2023 Jun 19;21:141. doi: 10.1186/s12915-023-01638-7 (PMC10280966; doi:10.1186/s12915-023-01638-7)
Supplement: Supplementary file 2 — Additional file 2: Fig. S1. Cross validation errors computed with ADMIXTURE v.1.3.0 by varying the number of superpopulations between K = 1 and 15. Fig. S2. Differentiation measured as Fstversus all chromosomal locations from OutFLANK analyses. Fig. S3. The workflow analysis for population genomics of the harlequin ladybird, Harmonia axyridis in the present study. Fig. S4. Demographic models of global invasion history of H. axyridis from an ancestral Asian source population tested in FSC26 between 3 superpopulations. Fig. S5. Demographic models of global invasion history of H. axyridis from an ancestral Eastern China source population tested in FSC26 between 4 superpopulations. Fig. S6. Goodness of fit estimates of the observed and estimated two dimensional site frequency spectra from our best fitting four-population model comprising asymmetric migration and exponential population size change. Fig. S7. Goodness of fit estimates of the observed and estimated two dimensional site frequency spectra from our best fitting three-population modelcomprising asymmetric migration and exponential population size change. [file 12915_2023_1638_MOESM2_ESM.docx]

**Global patterns of genomic and phenotypic variation in the invasive harlequin ladybird**

Hongran Li^1,2^, Yan Peng^2^, Yansong Wang^1^, Bryce Summerhays^3^, Xiaohan Shu^1^, Yumary Vasquez^3,4^, Hannah Vansant^3^, Christy Grenier^3^, Nicolette Gonzalez^3^, Khyati Kansagra^3^, Ryan Cartmill^3^, Edison Ryoiti Sujii ^6^, Ling Meng^1^, Xuguo Zhou^7^, Gábor L. Lövei^5^, John J Obrycki^7^, Arun Sethuraman^3,8*^ and Baoping Li^1*^

1 Department of Entomology, College of Plant Protection, Nanjing Agricultural University, Nanjing, P. R. China

2 Shenzhen Branch, Guangdong Laboratory of Lingnan Modern Agriculture, Genome Analysis Laboratory of the Ministry of Agriculture and Rural Affairs, Agricultural Genomics Institute at Shenzhen, Chinese Academy of Agricultural Sciences, Shenzhen, China

3 Department of Biological Sciences, California State University, San Marcos, CA, USA

4 Department of Life and Environmental Sciences, University of California, Merced, CA, USA

5 Department of Agroecology, Flakkebjerg Research Centre, Aarhus University; ELKH-DE Anthropocene Ecology Research Group, University of Debrecen, Hungary; Department of Zoology & Ecology, Hungarian University of Agriculture & Life Sciences, Godollo, Hungary

6 Empresa Brasileira de Pesquisa Agropecuária (Embrapa), Brasilia, DF, Brasil

7 Department of Entomology, University of Kentucky, KY, USA

8 Department of Biology, San Diego State University, CA, USA

* Co-corresponding authors, E-mails: Baoping Li: [lbp@njau.edu.cn](mailto:lbp@njau.edu.cn); Arun Sethuraman: [asethuraman@sdsu.edu](mailto:asethuraman@sdsu.edu)


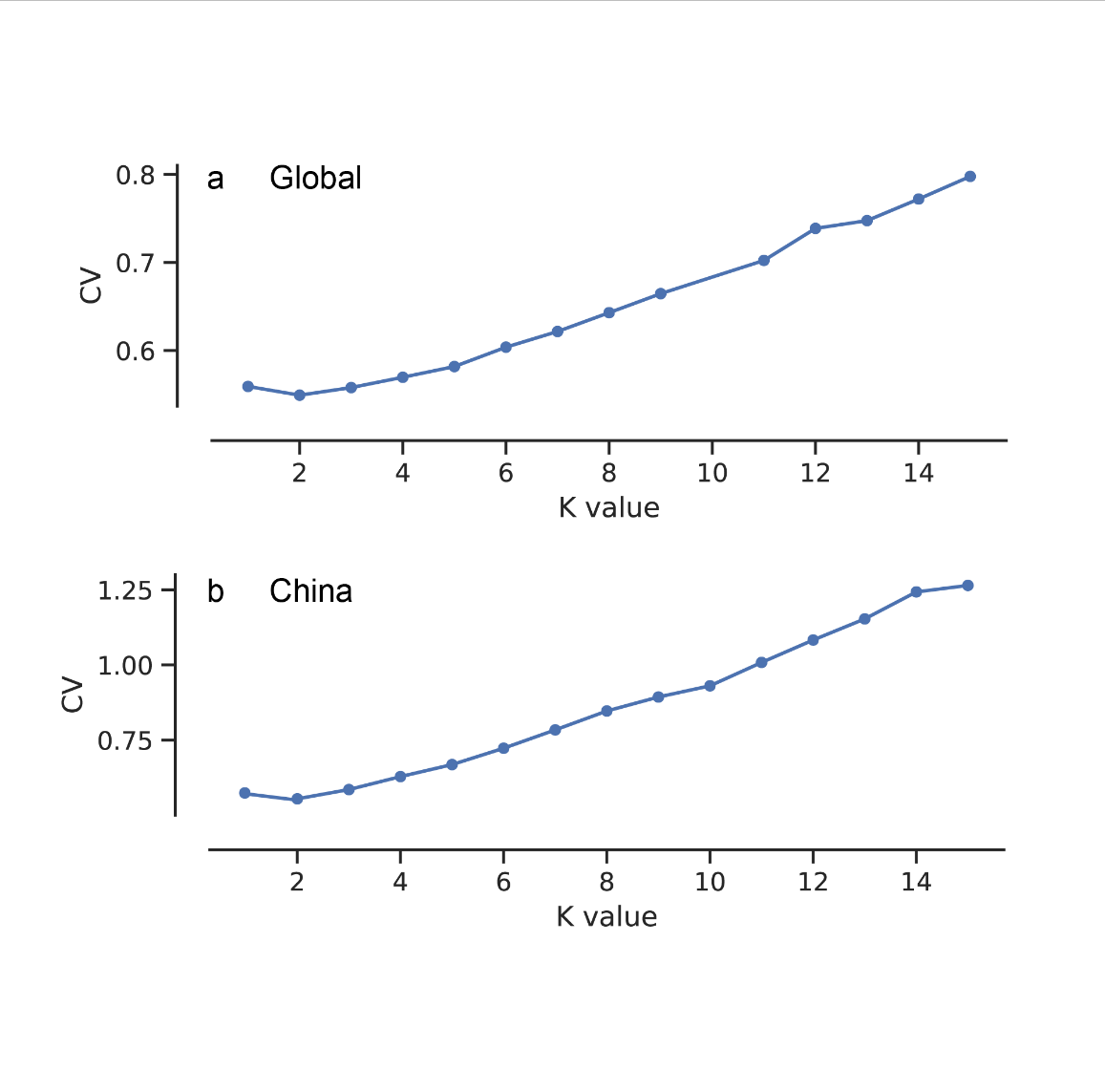


**Fig. S1** **Cross validation errors computed with ADMIXTURE v.1.3.0 by varying the number of superpopulations between K = 1 and 15.** **a.** Cross validation error for admixture of global populations. **b**. Cross validation error for admixture of Chinese populations.


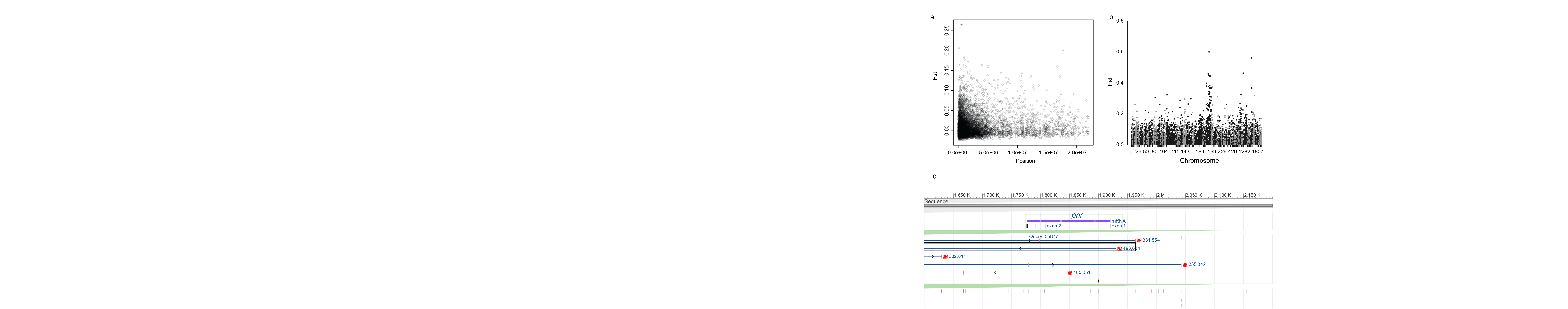


**Fig. S2 Differentiation measured as *F_st_* versus all chromosomal locations from OutFLANK analyses.** **a.** The single significant outlier locus located on scaffold 1381, mapping back to the Histone-lysine-N-methyltransferase SETMAR locus in the *H. axyridis* genome. Significance levels were assessed after correcting for false discovery rates using the FDR correction method implemented in OutFLANK. *F_st_* values were computed across all sampled populations. **b**. Weir and Cockerham’s *F*_ST_ across European, American, Western China, and Eastern China superpopulations, plotted along all 7824 loci analyzed across the *Harmonia axyridis* genome, showing significant outliers at scaffolds 1381 and scaffold 1185. **c**. NCBI BLAST queried homology of scaffold 1185 (region enclosing sites 629750-729750) to a region directly downstream of the *pannier* locus in the *H. axyridis* genome.


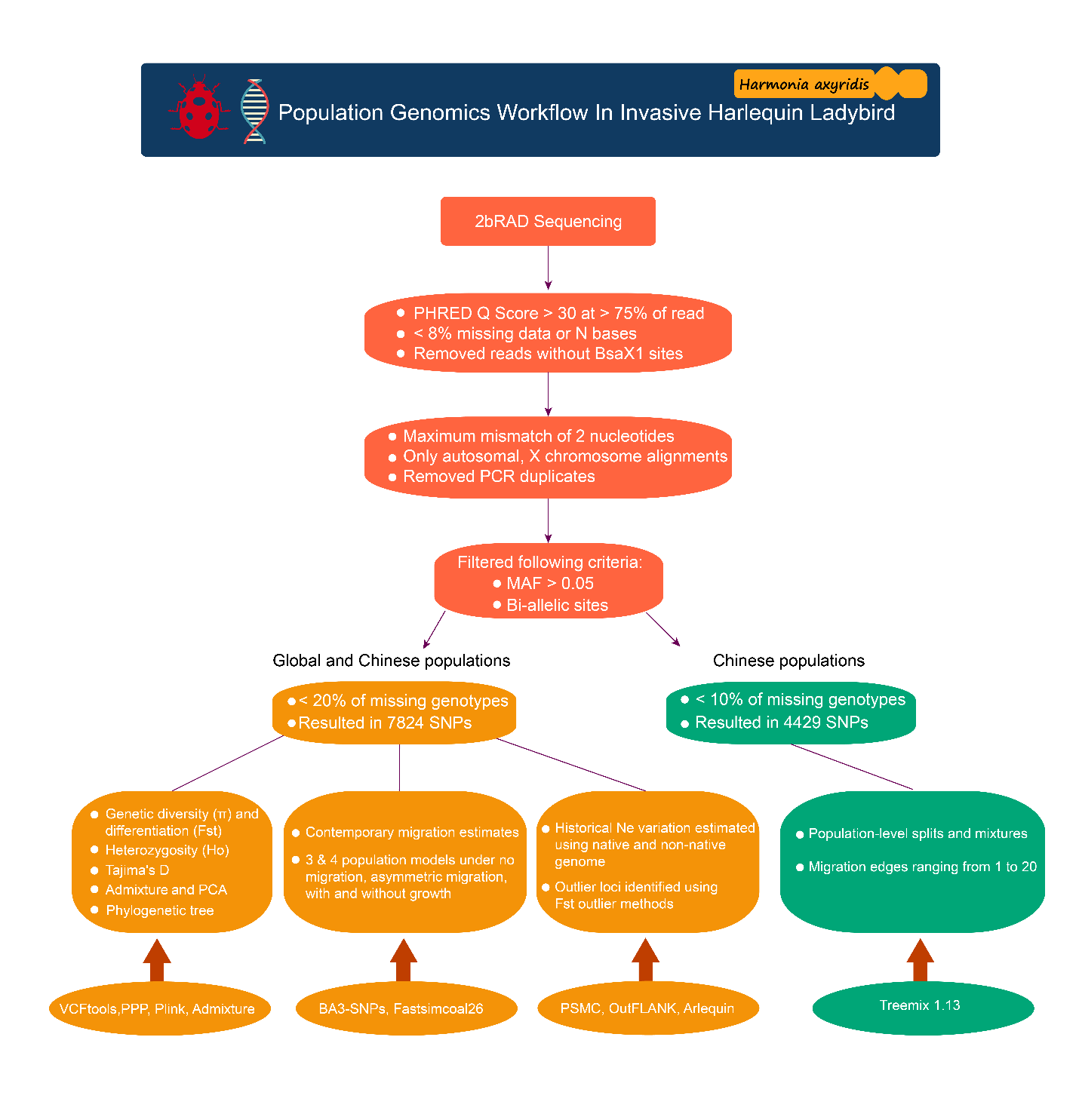


**Fig. S3 The workflow analysis for population genomics of the harlequin ladybird, *Harmonia axyridis* in the present study**


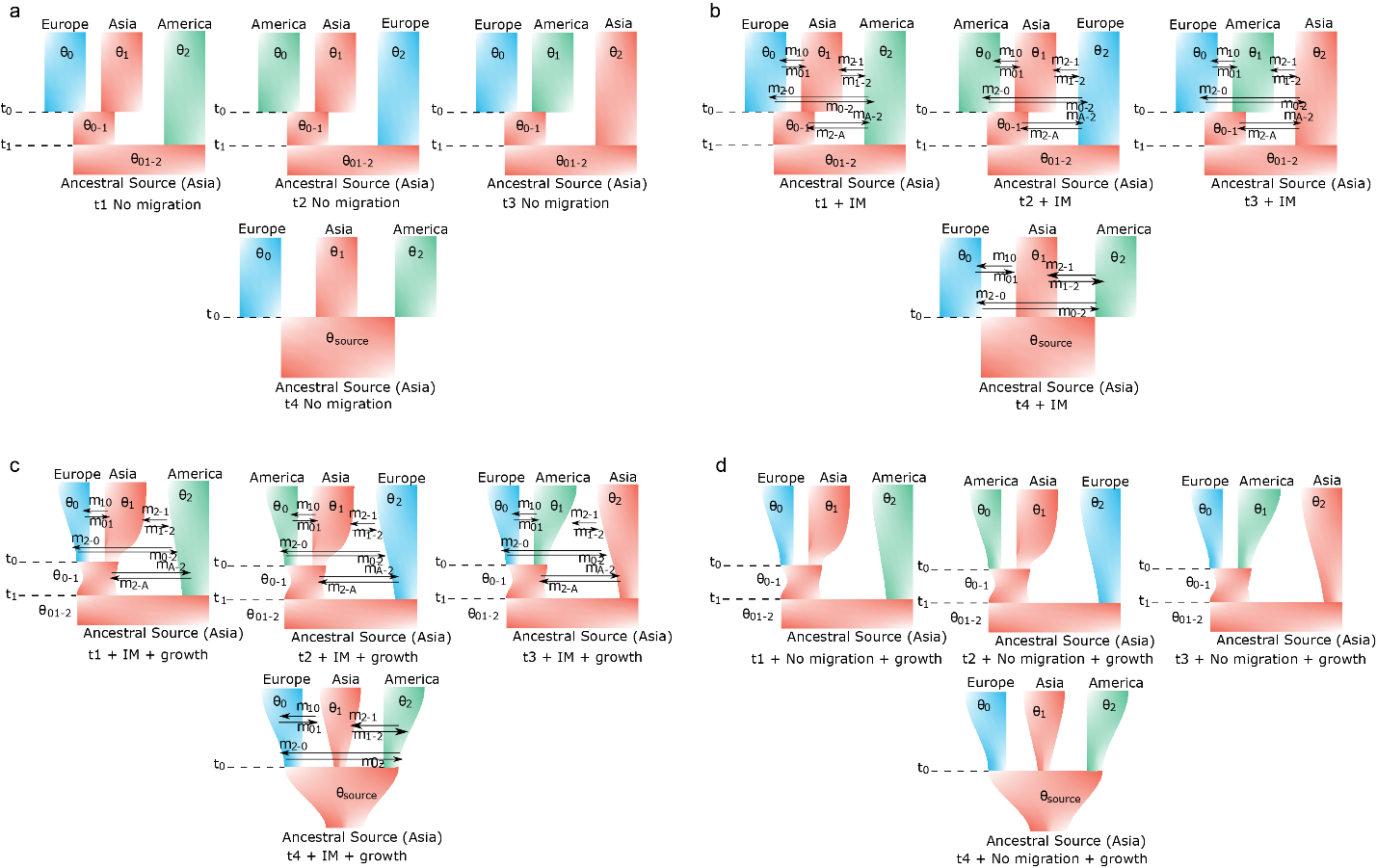


**Fig. S4** **Demographic models of global invasion history of *H. axyridis* from an ancestral Asian source population tested in FSC26 between 3 superpopulations (America, Europe, and Asia)**. Tested models include: (**a**) isolation without migration, (**b**) isolation with asymmetric migration, (**c**) isolation with asymmetric migration with changing population sizes, and (**d**) isolation without migration, with changing population sizes each under four topologies: (1) t1: ((Asia, Europe), MRCA1),USA) MRCA2, (2) t2: ((Asia,USA), MRCA1), Europe) MRCA2, (3) t3: ((Europe, USA), MRCA1), Asia)MRCA2, and (4) t4: (Asia, Europe, USA) MRCA. The best fitting model was estimated to be t1 + asymmetric migration and changing population sizes. θ = mutation scaled effective population sizes, m’s indicate migration rates, t’s are divergence times.


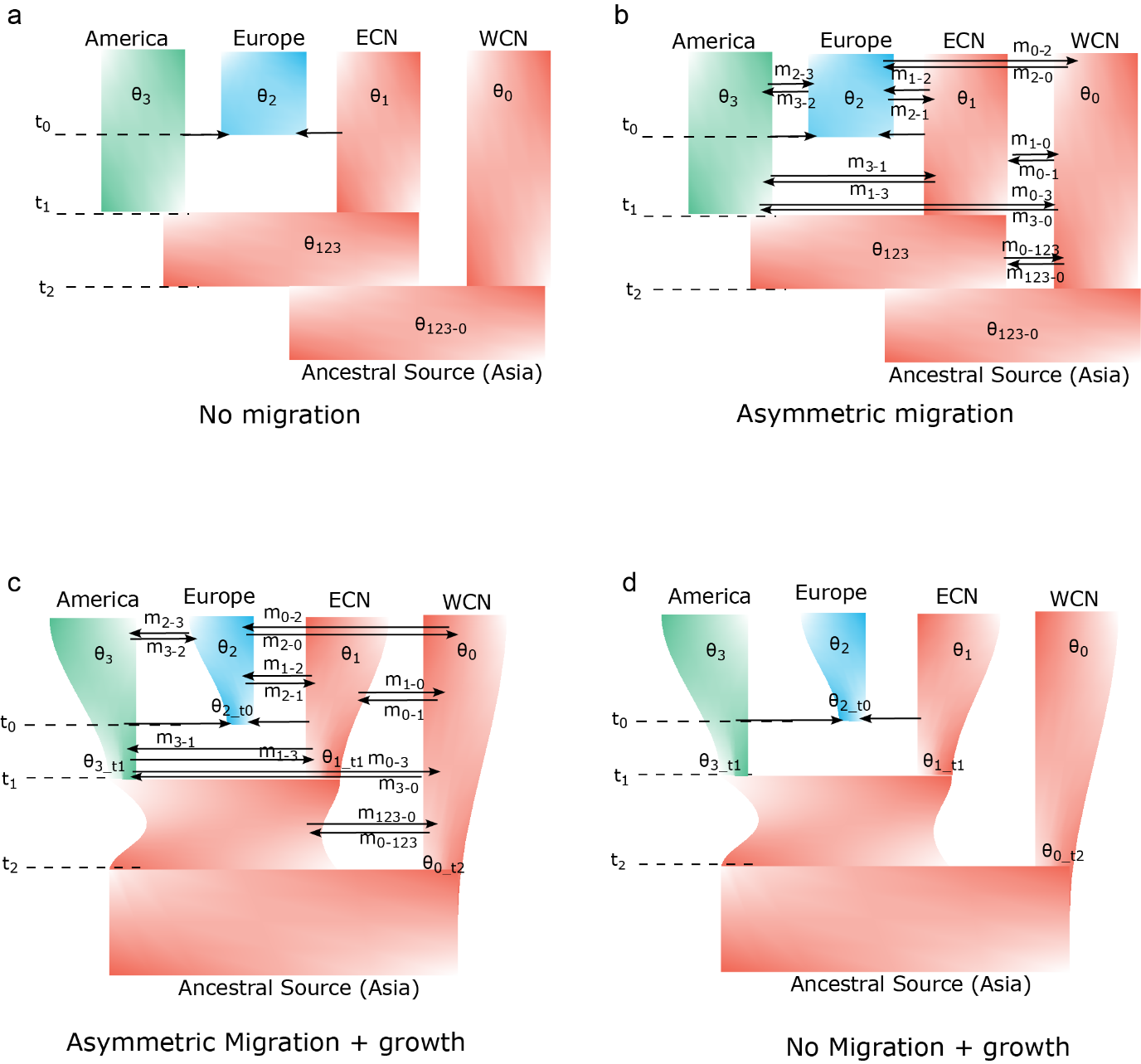


**Fig. S5** **Demographic models of global invasion history of *H. axyridis* from an ancestral Eastern China source population tested in FSC26 between 4 superpopulations (America, Europe, Eastern China and Western China).** Tested models include: (**a**) isolation without migration, (**b**) isolation with asymmetric migration, (**c**) isolation with asymmetric migration with changing population sizes, and (**d**) isolation without migration, with changing population sizes each under one topology: (1) t1: (((Eastern China + America = Europe:t0)t1),Western China) t2. The best fitting model was estimated to be t1 + asymmetric migration and changing population sizes. θ = mutation scaled effective population sizes, m’s indicate migration rates, t’s are divergence times.

**Fig. S6 (attached PDF file). Goodness of fit estimates of the observed and estimated two dimensional site frequency spectra from our best fitting four-population model (WCN, ECN, Europe, America)** **comprising asymmetric migration and exponential population size change.**

**Fig. S7 (attached PDF file). Goodness of fit estimates of the observed and estimated two dimensional site frequency spectra from our best fitting three-population model (WCN+ECN = China, Europe, America) comprising asymmetric migration and exponential population size change.**
